# *Apobec3* shows rapid evolution in house mouse subspecies and unusual hypermutation patterns of endogenous mouse mammary tumor viruses

**DOI:** 10.1128/jvi.01251-25

**Published:** 2025-09-16

**Authors:** Esther Shaffer, Guney Boso, Reza Sadjadpour, Venkat V. S. R. K. Yedavalli, Oscar Lam, Christine A. Kozak

**Affiliations:** 1 Viral Biology Section, National Institute of Allergy and Infectious Diseases 35037, Bethesda, Maryland, USA; 2 Department of Biological Sciences, Salisbury University 189366, Salisbury, Maryland, USA; 3 Graduate School of Biomedical Sciences, University of Massachusetts Chan Medical School 12262, Worcester, Massachusetts, USA; Icahn School of Medicine at Mount Sinai, New York, New York, USA

**Keywords:** mammary tumor viruses, *Apobec3 *hypermutation, adaptive evolution in mice, mouse *Apobec3 *evolution

## Abstract

Apobec3 (A3, apolipoprotein B editing complex 3) is a cytidine deaminase with broadly antiretroviral activity. A3 is packaged in virions and converts cytidines to uracil (C > U) in viral minus-strand DNA during reverse transcription, resulting in guanosine to adenosine (G > A) mutations in the viral plus strand. Mouse Apobec3 (mA3) mutagenizes numerous infectious retroviruses and some families of endogenous retroviruses. Here we show that endogenous mouse mammary tumor viruses, termed *Mtvs*, show a range of mA3 hypermutation from trace levels to an extreme example, *Mtv21,* that sustained a catastrophic, genome-wide reduction of G content to 10.8%; this level is associated with the contemporaneous generation of new preferred trinucleotide target sites by mutagenesis of consecutive cytidines. Although newly inserted proviruses have identical long terminal repeats (LTRs), mA3 can disproportionately generate G > A substitutions specific to the *Mtv* 3′ LTR. Individual hypermutated *Mtvs* display two different mA3 target site context preferences that correspond to those of the two mA3 alleles found among inbred strains of laboratory mice. *Mtv-*positive *Mus musculus* subspecies screened for these mA3 alleles unexpectedly revealed 20 distinct mA3 haplotypes with different combinations of replacement mutations at the 15 sites that distinguish the two inbred strain alleles, 11 of which show signatures of positive selection. These data show that mA3 deamination can have a major impact on the genomic integrity of *Mtvs*, and that mA3 is actively evolving within this single mouse species suggestive of high-impact genetic conflicts over a short evolutionary time frame.

**IMPORTANCE:**

The antiviral cytidine deaminase Apobec3 (apolipoprotein B editing complex 3) mutates retroviral DNA copies generated during new infections. Although such mutagenesis of replicating mouse retroviruses has been reported to be modest, here we show that many germline copies of mouse mammary tumor viruses (*Mtvs*) have sustained significant to massive levels of mouse Apobec3 (mA3) editing. mA3 hypermutation can also disproportionally affect one of the two otherwise identical viral long terminal repeats and can create new preferred target sites in the viral genome substrate by mutating successive cytosines. The edits in individual *Mtvs* correspond to the different target sequence preferences of the two inbred mouse strain mA3 alleles, but examination of allelic variation in wild mice of the species *Mus musculus* identified 18 additional variants and signatures of diversifying selection, a display of unusually rapid evolution within a single species over a short time frame.

## INTRODUCTION

Species susceptible to infectious retroviruses (XRVs) encode antiviral factors that target various stages of the retroviral life cycle (1). One such factor, Apobec3 (A3, apolipoprotein B editing complex 3), acts at a post-entry stage of virus replication. Orthologs are found in mammalian species, including human and mouse, where they have antiviral activity against multiple retroviruses (reviewed in reference 2). A3 is a cytidine deaminase that is incorporated into budding virions. During reverse transcription in subsequently infected cells, the virion-associated A3 catalyzes cytidine to uracil (C > U) deamination in the viral minus strand, resulting in guanosine to adenosine (G > A) mutations in the viral plus strand (3), which can impact viral fitness (reviewed in references 4–6). A3 also inhibits retroviral replication through a separate deamination-independent mechanism that inhibits reverse transcription (6–9).

A3 is a member of the APOBEC/AID gene family. The most extensively studied of the A3 orthologs is human *APOBEC3G* (hA3G), which is one of eight paralogs in primates (hA3A–H), most of which have antiviral properties (6, 10). In the mouse, the single *Apobec3* gene (mA3) has two allelic variants that differ in sequence, expression level- and splicing, all of which can influence its antiviral activity (7, 11–13). hA3G restricts multiple infectious viruses including HIV-1 and other lentiviruses and retroviruses, and mA3 also restricts HIV-1 as well as mouse mammary tumor viruses (MMTVs) and some mouse leukemia viruses (MLVs) (14–17). Mammalian genomes carry DNA copies of retroviral genomes inserted into host chromosomes termed endogenous retroviruses (ERVs); some human and mouse ERV families show evidence of hypermutation (18–20), and mA3 can also inhibit their retrotransposition (18). mA3 knockout mice are more susceptible to MMTV infection (14), and transgenics that overexpress members of the human AID/APOBEC family show increased levels of hypermutation and oncogenesis (21, 22). hA3G and mA3 both show evidence of positive selection in primates and rodents, indicative of an evolutionary history of genetic conflicts (13, 23, 24).

Several of the retroviruses targeted by A3 encode accessory factors that can antagonize A3. HIV-1 can avoid inhibition by hA3G through the action of the HIV-1 accessory protein, Vif (viral infectivity factor), which promotes ubiquitination of hA3G, leading to its proteasomal degradation (25–29). mA3 can also be inhibited by two MLV *gag* gene products: p50, which prevents packaging of mA3 (30), and glyco-Gag, which stabilizes the viral core preventing mA3 from having access to the viral genome (31).

While previous studies demonstrated that mA3 inhibits the infectivity and pathogenicity of MMTVs, proviruses acquired by MMTV-infected mice were observed to be relatively insensitive to mA3 hypermutation (14). However, our examination of 29 endogenous MMTVs, termed *Mtvs* (32), shows clear evidence of hypermutation ranging from marginal to massive and identifies examples of regional mutagenesis including four *Mtvs* with G > A mutations found in their 3′ but not 5′ LTRs (long terminal repeats). Individual hypermutated *Mtvs* exhibit different preferred target site contexts for G > A mutation that correspond to those of the two mA3 alleles found in the classical inbred strains (33). Inbred strains have only two mA3 alleles, but house mouse subspecies screened for these two mA3 alleles surprisingly carry 18 additional mA3 variants marked by different combinations of replacement mutations at the 15 sites that distinguish the two inbred strain alleles. Eleven of these sites are under positive selection, a rare display of remarkably rapid evolution in a single species.

## RESULTS

### Hypermutation of *Mtvs* by mA3

A set of 29 *Mtv* ERVs extracted from the 17 sequenced genomes of classical inbred mouse strains and wild mouse taxa (32) were assessed for editing by mA3. In the absence of identifiable progenitors for these *Mtvs*, we aligned the *Mtvs* of laboratory strains and *Mus musculus* subspecies with the nondefective *Mtv1* as shown for *Mtv21* (Data File S1); among the nondefective *Mtvs* and infectious MMTVs, *Mtv1* was the closest match for the *M. musculus* MMTVs. The seven 2-LTR *Mus spretus Mtvs* are not as closely related to the laboratory mouse MMTVs (32), so they were instead aligned with a consensus sequence for this group. Because of extensive variability in the C-terminus of the LTR-encoded *sag* gene, alignments extended from the beginning of *gag* through the N-terminal two-thirds of the *sag* gene in the 3′ LTR, a segment of 7,924 bp in *Mtv1*.

Table 1 describes the mA3 mutation profile for the 21 *Mtvs* that have coding sequence. *Mtvs* were evaluated for G > A, C > T, and A > G mutations. Hypermutation is recognizable by a clear excess of G > A mutations. mA3 editing tends to be minus-strand specific, so it is marked by more G > A than C > T changes in the viral plus strand, and it is unidirectional, with a preponderance of G > A vs A > G mutations. The *Mtvs* listed in Table 1 show a wide range of hypermutation with over half showing at least a twofold excess of G > A mutations. Hypermutation was not detected in the six *Mtv* solo LTRs in the sequenced mouse genomes (Table S1), but there are some examples among the *Mtv env, pol,* and *sag* sequences cloned from wild mouse DNAs (Table S2). While random mutation would be expected to produce equal numbers of the different mismatches, the 5′ end of *Mtv61* has almost twice the number of A > G as C > T mismatches and a greater number of A > G than G > A mismatches, which is suggestive of mutagenesis by the adenosine deaminases that act on RNA (ADAR) enzymes. ADAR1 has been found to mutate HIV-1 (34), so while this is a possible explanation for the mutation pattern in the 5′ end of *Mtv61*, the sequence context around these A > G mutations does not show the pattern associated with ADAR mutations (35).

**TABLE 1:** Evidence of mA3 editing of *Mtvs* found in sequenced mouse genomes

Subspecies	*Mtv*	%G	%A	Mismatches^*a*^					Runs of G > A^*b*^			
				G > A	C > T	A > G	Total	%G > A/ Total	2	3	4	5
*musculus*	*Mtv1*	20.8	26.7									
	*Mtv21*	10.8	41.8	943	57	34	1,158	82.6	128	32	12	3
	*Mtv61*	18.5	33.9	383	139	177	997	38.8	32	6	4	1
	*Mtv61*-5′	21.0	33.0	107	70	131	479	22.3	3			
	*Mtv61*-3′	14.7	35.2	276	69	46	518	54.0	29	6	4	1
	*Mtv57*	20.7	31.8	172	31	36	307	56.4	16			1
	*Mtv7*	21.8	30.7	98	66	78	366	26.8	2			
	*Mtv23*	21.3	31.2	87	19	16	152	57.2	5			
	*Mtv17*	21.5	31.0	87	19	16	152	57.2	5			
	*Mtv62*	21.6	31.0	88	12	16	135	61.5	2			
	*Mtv3*	21.7	30.9	86	45	46	267	32.2	3			
	*Mtv55*	21.8	30.7	77	41	40	238	32.3	1			
	*Mtv9*	22.0	30.6	75	61	53	291	25.8	2			
	*Mtv11*	22.0	30.5	46	13	16	102	45.1	1			
	*Mtv8*	22.0	30.4	43	21	28	136	31.6	1			
	*Mtv13*	20.9	26.5	11	12	17	70	15.7				
	*Mtv56*	22.1	30.9	9	8	6	32	28.1				
	*Mtv6*	23.1	29.6	4	6	10	33	12.1				
*spretus*	*Mtv32*	22.8	30.6	13	1	5	29	44.8				
	*Mtv34*	22.2	30.5	62	35	54	246	25.2				
	*Mtv35*	20.2	26.5	33	16	21	118	28.0	1			
	*Mtv36*	20.7	26.9	18	8	8	62	29.0				
	*Mtv37*	20.7	26.9	19	6	11	51	37.3				
	*Mtv38*	22.2	30.2	55	42	44	223	24.7	1			

^*a*^ Mismatches were based on alignments of full-length *M. musculus Mtvs* (32) with *Mtv1*, and the *M. spretus Mtvs* with a group consensus sequence. The differentially mutated 5′ and 3′ halves of *Mtv61* were analyzed separately. G > A (guanosine to adenosine), C > T (cytosine to thymidine), A > G (adenosine to guanosine).

^*b*^ Numbers of runs of two to five consecutive G > A mutations.

As shown in Table 1, A3 activity is also marked by mutation of consecutive cytosines (C tracts) producing runs of G > A mutations in the viral genome (36, 37). A3 mutagenesis can also create premature stop codons, disable ATG start sites, and produce nonsynonymous mutations (38). All but one of the full-length *Mtvs* carry at least two open reading frames (ORFs) (Table S3). The dysfunctional coding regions in these *Mtvs* collectively contain 36 unique stop codons, all of which are mutated tryptophan codons (TGG) (Table 2). Four hypermutated *Mtvs* were analyzed for amino acid substitutions due to G > A mutations; for most genes, over half of the G > A mismatches alter the amino acid sequence (Table S4).

**TABLE 2:** Premature protein termination codons in 12 full-length *Mtvs*

Viral gene	*Mtv*	Site position^*a*^													
*gag*	21	45		285		457	563								
	57		210		402	457		624							
	62				402										
*pro*	21	162	231	253											
*pol*	21	20	61	75	108	150	269	352	623	642	719	801	809		
	17	20	61				269					801			
	23 61 62	20	61				269					801			
											719	801	809		
			61		108	150									
*env*	21	57	116	201	204	241	251	273	309	314	321	436	562	573	621
	17	57									321			573	
	23	57									321			573	
	3	57												573	
	9	57											562		
	7	57													
	57							273			321		562	573	
	61										321		562	573	621
	62													573	
	11													573	
	55														621
	8														621

^*a*^ Positions are relative to the *gag* and *env* start sites and the frameshift sites for *pro* and *pol*.

### Individual *Mtvs* that are ultra-edited or show hypermutation gradients

**Fig 1:**
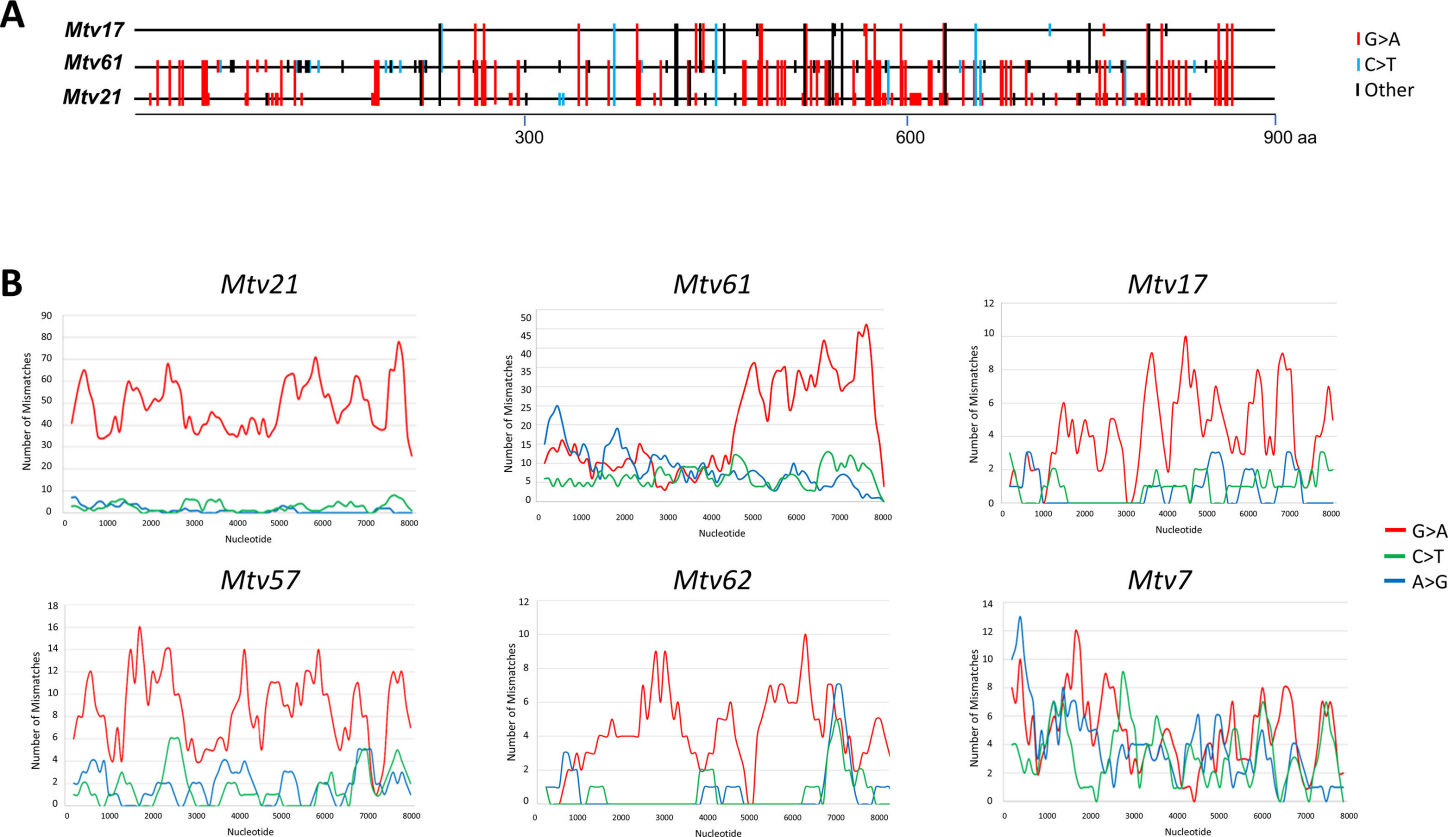
Hypermutation in *Mtv* genomes. (**A**) Sequence mismatches in three *Mtv sag* genes in an alignment of a segment of *sag* from the start codon to the beginning of the variable domain near the C-terminus. G > A, C > T, and other substitutions are identified by colored lines, and extended lines represent shared substitutions. (**B**) Gradients of G > A mutations across the genomes of six *Mtvs*. Sliding window analysis of 400 bases with 100 step size is shown for the indicated *Mtvs*. The assessed alignment extends from the *gag* ATG (position 1) to the variable domain of the 3′ encoded LTR.

*Mtv21*, found in the sequenced NZO/HILtJ strain genome, and *Mtv61,* from the wild-derived CAST/EiJ mouse, carry an exceptional number of G > A mismatches (Table 1, Fig. 1A; Data File S1). Because mA3 hypermutation preferentially targets specific di- or trinucleotides (39), there are multiple shared mismatches in these *Mtvs* as illustrated for three *Mtv sag* genes (Fig. 1A). *Mtv21* has an overall proviral G content of only 10.8% (8.3% in *env*) and a corresponding increase in A content to 41.8% (Table 1). This provirus also shows multiple consecutive G > A substitutions and is the only one of these *Mtvs* that has no ORFs. This degree of hypermutation is specific to the *Mtv21* sequence as 4 kb of cellular DNA flanking this *Mtv* show no such pattern and are >99.6% identical to the homologous segment in the C57BL/6 (B6) reference genome (Data File S2). This extreme degree of editing of an embedded provirus is thus not the cumulative result of ongoing mutagenesis that spares the immediately flanking DNA.

A sliding window analysis of hypermutation was done for the highly mutated *Mtvs*, *Mtv17, 21, 57, 61,* and *62,* and for one with little evidence of editing, *Mtv7. Mtv21, 17, 57,* and *62* show hypermutation across their genomes, although the most highly edited one, *Mtv21,* displays less extreme local variability than the others (Fig. 1B). In contrast, *Mtv61* shows a dramatic hypermutation gradient drop with a sharp decrease in G > A substitutions throughout the 5′ half of its genome, a gradient that does not resemble the local variations in the mutational gradients produced by hA3G in HIV-1, which peak 5′ to its two PPTs (polypurine tracts) (38, 40).

The extreme levels of hypermutation in *Mtv21* and the 3′ portion of *Mtv61* were compared to a catalog of ultra-edited ERVs (41) (Table 3). These two *Mtvs* showed the highest number of G > A mutations per 1,000 bp. With respect to their total mutational profiles, these *Mtv* levels are comparable to the highest reported examples of ultra-edited ERVs, found in zebra finches, and for IAPs (intracisternal A-type particles) in mice. Among the hypermutated proviruses generated in virus-infected cells, the highest level of hypermutation has been reported for Vau-O HIV-1 (42, 43), but *Mtv21* shows a greater number of G > A mismatches (Table 3). High levels of HIV-1 editing have also been reported in human patient peripheral blood mononuclear cells (PBMCs), measured as saturation of the dinucleotide target sequence of hA3G (44).

**TABLE 3:** Comparison of the extensively hypermutated *Mtv21* and *Mtv61* with previously described ultra-edited ERVs and XRVs^*a*^

Type	Organism	ERV/XRV^a^	Length (bp)	G > A (#)	Total mismatches	% G > A/ Mismatches	G > A mismatches per 1,000 bp	Reference
ERV	Mouse	*Mtv21*	7,924	957	1,158	82.6	120.8	Present study
		*Mtv61*	7,923	387	997	38.8	48.8	
		*Mtv61*-3′	3,450	280	518	54.1	81.2	
	Zebra finch	TguERVl1_I-int	5,486	392	475	82.53	71.5	(41)
		TguLTRK1_1	6,173	353	440	80.23	57.2	
	Mouse	MuRRS-int	4,657	199	225	88.44	42.7	
		IAPEz-int	6,382	198	233	85.34	31.0	
	Human	HERVK-int	5,375	148	205	72.2	27.5	
	Rat	NICER2_Ra-int	7,513	93	100	93	12.4	
XRV		HIV-1_VAU_	9,330	679	722	94	72.8	(43)

^*a*^ ERV, endogenous retrovirus; XRV, exogenous retrovirus.

### Multiple *Mtvs* carry G > A mutations unique to their 3′ LTRs

**Fig 2:**
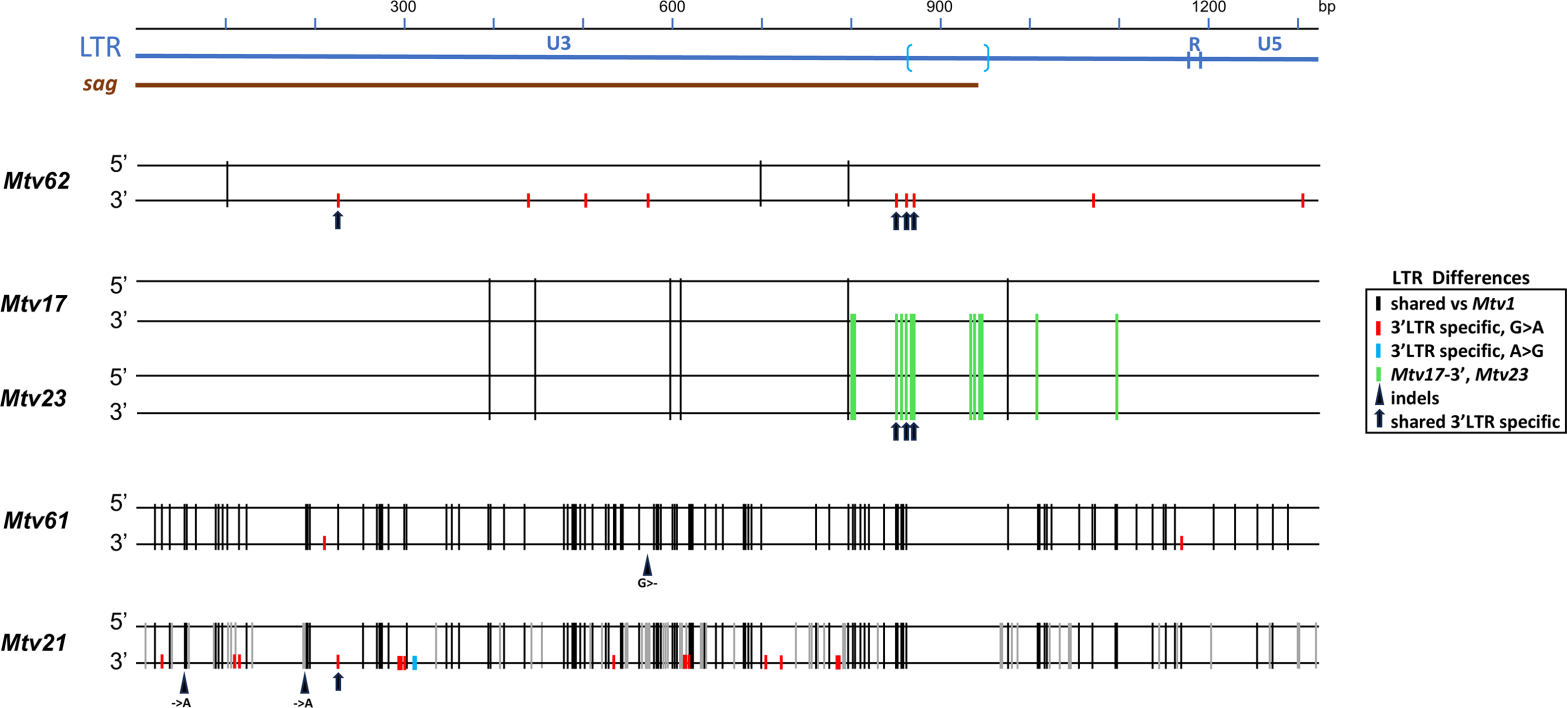
*Mtvs* with 3′ LTR specific G > A mutations. At the top is an LTR diagram using numbering based on *Mtv1* and showing the U3, R, and U5 regions. The *sag* gene coding region is identified by a brown line and brackets marking the highly variable region. For each *Mtv*, the 5′ and 3′ LTRs were aligned with each other and with *Mtv1* to identify shared or distinctive hypermutations. Vertical lines identify substitutions that are shared by the two LTRs relative to the *Mtv1* reference sequence (black), 3′ LTR-specific (red, blue) or common to the two *Mtv23* LTRs and the 3′ LTR of *Mtv17* (green). Mismatches relative to *Mtv1* could not be reliably identified in the bracketed segment. Triangles identify single base deletions or insertions. The three 3′ LTR mismatches shared by *Mtv62* and *Mtv17* and *23,* and the one shared by *Mtv62* and *Mtv21* are identified by arrows.

When ERVs are formed, the flanking LTRs are identical, but over time, they independently acquire mutations and diverge in sequence (45). *Mtvs* are relatively recent additions to the mouse genome (0.5 million years ago [MYA]) (32, 46–48) and so would be expected to show minimal LTR variation. In fact, 17 of the 21 2-LTR *Mtvs* have identical LTRs. The four exceptions (*Mtv17, Mtv21, Mtv61, Mtv62*) show clear evidence of mA3 mutations throughout their genomes (Table 1). The two LTRs in each of these *Mtvs* have multiple shared hypermutations but also respectively carry 13, 16, 2, and 9 nucleotide substitutions that distinguish the two LTRs (Fig. 2). All but one of these mismatches are G > A mutations that are unique to the 3′ LTR; the single exception is an A > G mutation in the *Mtv21* 3′ LTR. The LTR discrepancies in these four *Mtvs* are not sequencing errors, as identical copies of *Mtv17* are carried by the sequenced genomes of eight inbred strains (129, AKR, C57BL/6, CBA, DBA, FVB, LP, NOD) (32), and resequencing both LTRs of the other three *Mtvs* confirmed these LTR discrepancies.

That these additional mutations are induced by mA3 is supported by the fact that there are six examples of consecutive G > A mutations among the 3′ LTR-specific G > A mismatches, three each in *Mtv21* and *Mtv17*, a hallmark of A3 hypermutation, and that among the 39 G > A mutations in these four *Mtvs*, the same three sites are mutated in *Mtv62* and *Mtv17*, and the same one in *Mtv62* and *Mtv21*, consistent with A3 target context specificity (33, 39).

All but two of the mutations unique to the 3′ LTR are in U3, which constitutes the bulk of the *Mtv* LTR and also encodes Sag and carries transcription factor binding sites. Functionality of the 3′ LTR can have consequences for the host in that production of Sag can influence susceptibility to MMTV infection (49), and the LTR promoter can alter expression of nearby host genes. While the *sag* genes of *Mtv61* and *21* are defective due to indels that generate frameshifts, *Mtv62* and *Mtv17* have *sag* ORFs, and the added 3′ LTR mutations produce seven and eight amino acid substitutions, respectively (Table S4). The three shared mismatches in *Mtv17* and *62* lie in the C-terminus of *sag* (Fig. 2), which is associated with Sag specificity and the ability to restrict exogenous MMTV infections (49, 50), so persistence of these *Mtvs* could be due to a selectable fitness benefit to the host. It is also possible that mutations in transcription factor binding sites in U3 have consequences for host gene regulation. For example, one of the two MAF1 binding sites (51) is modified by 3′ LTR mutations in *Mtv21* (GGGGCAAG > GAAGCAAA), as is an NF1 site (52) in *Mtv17* (TTGGAACTTATCCAA > TTAGAACTTATCCAA), but the three GRE sites and the two OCT1 sites (52) are identical in all four *Mtvs* and the *Mtv1* reference.

Such G > A differences in proviral LTRs have not been previously reported for other retroviruses, so we examined the LTRs of MLV ERVs, acquisition of which roughly coincided with that of *Mtvs* (32, 53). One MLV, *Mpmv5*, has four G > A mutations specific to its 3′ LTR (Fig. S1), suggesting this phenomenon is not specific to *Mtvs*. Nine additional MLV ERVs have single mutations that distinguish their LTRs, three of which carry 3′ LTR-specific G > A substitutions (*Pmv23, Mpmv7, Mpmv46*).

One of the *Mtvs* with different LTRs, *Mtv17*, is virtually identical in sequence to *Mtv23*. These two ERVs share the same overall hypermutation pattern and carry the same set of other genome-wide mutations (Tables 1 and 2). *Mtv17* and *Mtv23* map to different chromosomes, have different target site duplications, and are carried by different subsets of inbred strains (32). The two *Mtv23* LTRs, however, are identical and have the same hypermutation pattern as the *Mtv17* 3′ LTR, including the 13 additional G > A changes not found in the *Mtv17* 5′ LTR (Fig. 2). This suggests that *Mtv23* is a copy of *Mtv17* with both LTRs derived from the *Mtv17* 3′ LTR.

### Target site context dependence

The A3 proteins from different species target signature di- or trinucleotides (39). Two target site contexts have been identified in mA3-edited HIV-1 or MLV proviruses in newly infected cells and for various mouse ERVs, including IAPs, the MusD retrotransposons, and MLVs (18, 19, 33, 39). These site context preferences, with the target site underlined, are XTC or T(T/C)C, or, as seen on the plus strand, GAX or G(G/A)A. While mA3 has therefore been described as less stringent than other A3s in its preferred target site, the observed sites have been identified as the target site preferences of the two allelic variants of mA3 found in the inbred strains BALB/c (mA3^BALB^) and C57BL/6 (mA3^B6^). mA3^B6^ has a stronger preference for T(T/C)C, whereas mA3^BALB^ prefers XTC (33).

**Fig 3:**
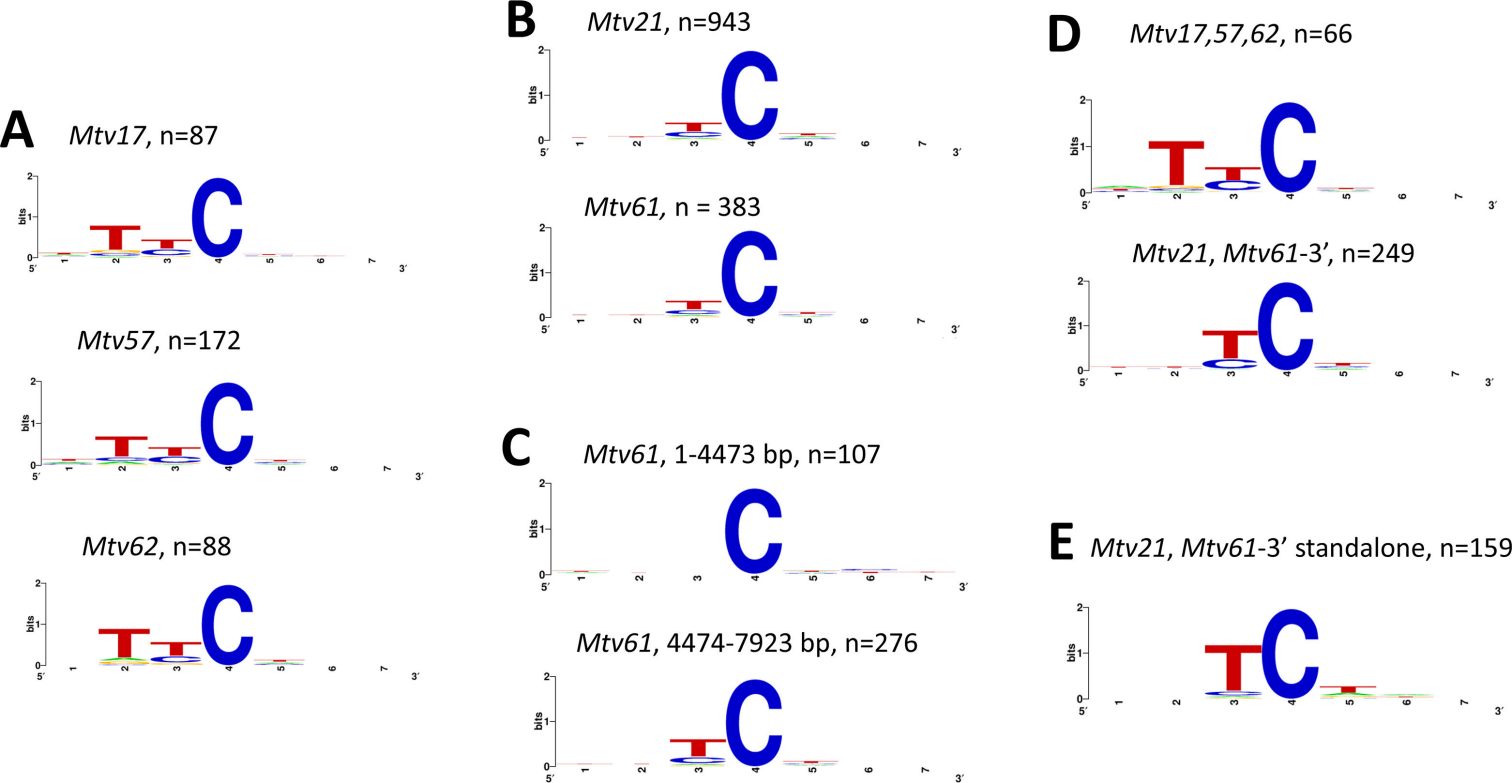
Target site analysis of hypermutated sites in each of six *Mtv*s using Weblogo. (**A**) Three *Mtvs* with a clear excess of G > A substitutions showing mA3^B6^-like target preferences. (**B**) The two most highly edited *Mtvs* showing mA3^BALB^-like preferences. (**C**) The 5′ and 3′ halves of *Mtv61*. (**D**) Shared C > T substitutions in the indicated *Mtvs*. (**E**) The subset of shared C > T substitutions of *Mtv21* and *Mtv61*-3´ with no mutations in the −1 or −2 positions relative to the reference sequence.

We separately examined the context dependence for the set of edits in each of five highly hypermutated *Mtvs* (Fig. 3; Fig. S2). While the most common target of hypermutation is TTC in all five *Mtvs* (GAA on the plus strand) (Fig. S2), different nucleotides at the −1 and −2 positions influence the likelihood of mutation. Three *Mtvs* (*Mtv17, 57, 62*) showed an mA3 target site preference for T(T/C)C (Fig. 3A; Fig. S2A), while two *Mtvs (Mtv21, Mtv61*) showed an XTC preference (Fig. 3B; Fig. S2B). We also separately examined the target site preferences in the 3′ and 5′ halves of *Mtv61*. The 3′ half, with a high level of hypermutation, shows a preference for XTC, whereas the limited hypermutation of the 5′ end shows a marginal preference for T(T/C)T (Fig. S2C). This apparent mid-virus transition in target site difference in *Mtv61*, together with the unusually abrupt change in the extent of hypermutation across its genome, suggests that *Mtv61* may be a recombinant.

Since target site specificity results in shared mutations in different proviruses as illustrated in Fig. 1A, we re-examined these two subsets of edited *Mtvs* using only the shared sites in each set to eliminate weak targets and mutations unrelated to mA3 editing. *Mtv17, 57,* and *62* were scored for sites shared by any two of these three proviruses, and analysis of the shared G > A sites in *Mtv21* and *Mtv61* included only the segment spanning the hypermutated 3′ end of *Mtv61*. For both subsets, this produced stronger associations with the same two target sites, marked by the enhanced importance of T at position −1 in *Mtv21* and *61,* and for T, at position −2 in *Mtvs 17, 57,* and *62* (Fig. 3D; Fig. S2D). This is consistent with the conclusion that the two sets of *Mtvs* were altered by the different inbred strain mA3 variants.

C tracts in the minus strand can sustain consecutive mA3 mutations, most notably seen here for *Mtv21* (Table 1, Data File S1)*,* but C tracts do not contain either of the preferred target sites for mA3. Our initial target site analysis was based on the assumption that each mutation is independent, and that the target site is defined by the reference sequence. However, the concentration of mutations in C tracts suggests that ongoing mutagenesis may create new, immediately available target sites by converting CCC trinucleotides to mA3-susceptible TTC, CTC, or TCC (54). Because of the unusually high number of C tract mutations in *Mtv21* and *Mtv61,* seen as consecutive plus strand G > A mutations*,* we re-analyzed mutations in these *Mtvs* excluding those sites with C > T mutations in the −1 and −2 positions for shared mutations (Fig. 3E) or for *Mtv21* alone (Fig. S2E). These “standalone” mutations show a target site profile with enhanced involvement of T at the −1 position. This suggests that using the unaltered reference sequence to define target site context distorts the analysis because mA3 site selection can be affected by contemporaneous, adjacent C > T mutations. This accounts for the clustering of hypermutations, as well as the increased overall level of mutagenesis.

Thus, different *Mtvs* show one of two mA3 target site preferences, suggesting that they have been deaminated by different mA3 variants, and that progressive mA3 mutations can create new target sites, generating runs of hypermutations.

### Genetic variants of mA3 in mouse strains and taxa

We screened classical inbred mouse strains and taxa harboring *Mtvs* for the presence of the two inbred strain mA3 variants, mA3^BALB^ and mA3^B6^. These variants have three distinguishing features: sequence polymorphisms altering 15 residues, the incorporation or exclusion of exon 5, and the presence or absence of an inserted MLV LTR (7, 13, 55, 56) . These alleles differ in their ability to inhibit MMTV- and MLV-induced disease and virus replication in mice (7, 11, 12, 14). While it is not entirely clear how each of these features contribute to this inhibition, mA3^B6^, which blocks MLV replication and disease induction, includes the MLV LTR but not exon 5 (7, 11).

Among the 12 inbred strains with sequenced genomes, we identified mA3^B6^ in two, NZO/HILtJ and B6, while mA3^BALB^ was found in 10 strains based on sequence polymorphisms, the presence/absence of the MLV LTR, and the two sequence variants linked to exon 5 inclusion (Table S5). Direct sequencing of an additional 32 strains for the coding sequence variants and the LTR identified mA3^B6^ in five and mA3^BALB^ in the rest (Table S5). There was no correlation between the mA3 allele of a given mouse strain and the hypermutation profiles of its *Mtvs*, which is not unexpected, as most *Mtvs* predate strain origins (32), and because strain development often involved deliberate or inadvertent cross-breeding (57) that could introduce the alternative mA3 allele as well as *Mtvs* that would then be shared by strains carrying different mA3 alleles. For example, *Mtv21* is carried by NZO/HILtJ (mA3^B6^), as well as NZW/LacJ and NZM2410/J (mA3^BALB^) (Table 4).

**TABLE 4:** *Apobec3* alleles carried by inbred strains carrying shared hypermutated *Mtvs*

*Mtv*	Target site (no. of G > A mismatches)^*a*^	mA3 allele in *Mtv* positive strains	
		mA3^B6^	mA3^BALB^
21	XTC (943)	NZO/HILtJ	NZM2410/J, NZW/LacJ
57	T(T/C)C (172)	RF/J, NZB/BINJ	C58/J, I/LnJ, LP/J, LT/SvEiJ
23	T(T/C)C (87)	RF/J	A/J, AKR/J, NON/ShiLtJ ,PL/J
17	T(T/C)C (86)	C57BL/6J, NZO/HILtJ, RF/J, NZB/BINJ	129X1/SvJ, AKR/J, C57BR/cdJ, C57L/J, C58/J, CBA/J, DBA/2J, F/St, FVB/NJ, I/LnJ, LG/J, LP/J, LT/SvEiJ, MA/MyJ, NFS/N, NOD/ShiLtJ, NON/ShiLtJ, NZW/LacJ, PL/J, SM/J, SWR/J, TALLYHO/JngJ
3	T(T/C)C (86)	NZB/BINJ, NZO/HILtJ	C58/J, F/St, KK/HIJ, NOD/ShiLtJ, NON/ShiLtJ, NOR/LtJ, NZW/LacJ, PL/J
7	T(T/C)C (98)	NZO/HILtJ, RF/J, NZB/BINJ	C58/J, DBA/2J, I/LnJ, LT/SvEiJ, NFS/N, NON/ShiLtJ, SEA/GnJ, SM/J, SWR/J,
55	– (77)		129X1/SvJ, LP/J, KK/HIJ
9	– (75)	C57BL/J, NZB/BINJ	129X1/SvJ, AKR/J, BALB/cJ, C57BR/cdJ, C57L/J, LG/J, LP/J, NOR/LtJ, SEC/1ReJ

^*a*^ Target site preferences determined by sequence logos generated by Weblogo. –, no clear preferences.

**Fig 4:**
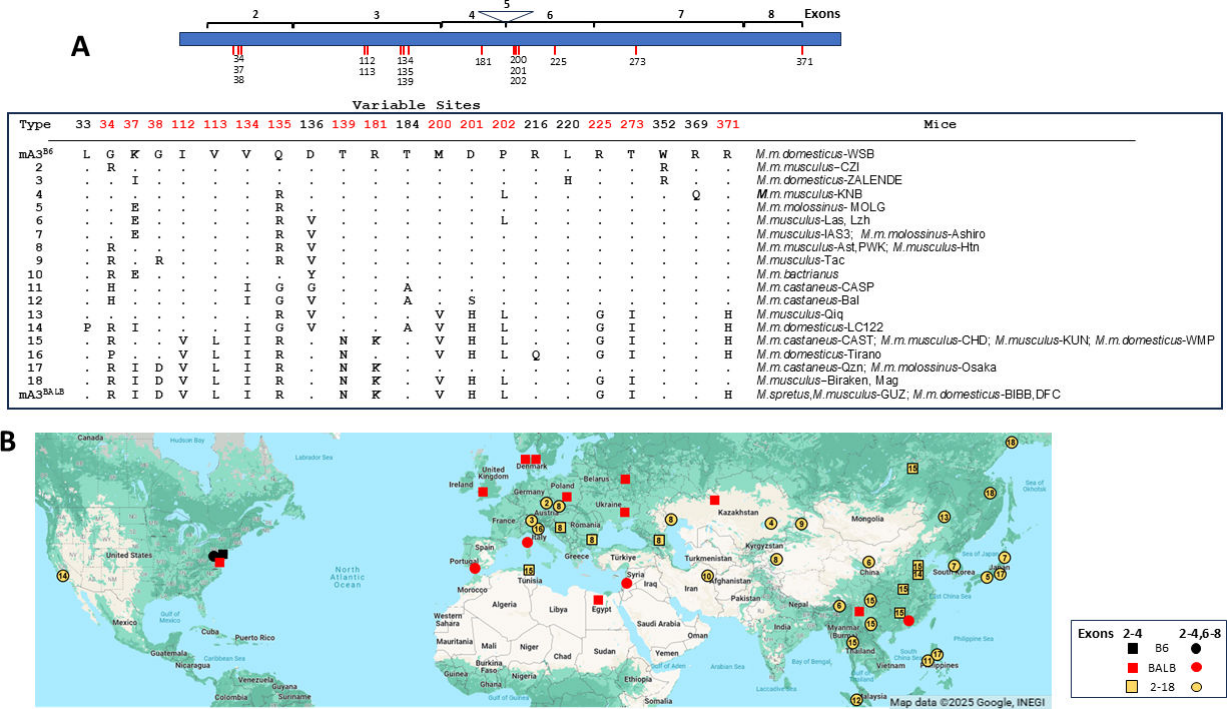
Twenty haplotype variants of mA3 in wild mice. (**A**) A diagram of the mA3 protein showing the positions of the sequenced exons 2–4 and 6–8. Exons 2–4 encode the active CD, and the exon 6–8 CD is inactive. Exon 5 is variably present. Red tick marks identify the 15 replacement mutations that distinguish the mA3^B6^ and mA3^BALB^ proteins. The chart shows sequence variations at 22 sites that define 18 novel haplotype variants in various *M. musculus* and *M. spretus* wild mice relative to mA3^B6^ and mA3^BALB^. (**B**) Map showing the geographic locations of the trapping sites for mice typed for mA3 sequence type. Genes equivalent to the two alleles found in laboratory mice are in red and black, with novel alleles in yellow. Circles represent mice sequenced for the exons covering both mA3 CDs, while squares represent sequences for just the active CD. Numbers identify the haplotypes described in panel A.

Selected wild-derived and wild-trapped *Mus musculus* mice were screened to determine the distribution of the two known mA3 variants in natural populations of the progenitor subspecies of the classical inbred strains. PCR amplified genomic segments containing exons 2–4 and 6–8 from 31 wild mice unexpectedly revealed significant sequence variation with polymorphisms almost exclusively affecting the 15 sites that distinguish mA3^B6^ and mA3^BALB^ (Fig. 4A). Different combinations of these polymorphisms produce 18 novel haplotypes of mA3.

The mA3^BALB^ sequence variant was identified in three of the 30 *M*. *musculus* mice and 11 of 19 additional wild mouse DNAs sequenced only for exons 2–4, which encode the active cytidine deaminase domain (CD) of mA3 (Fig. 4A; Fig. S3). Like other A3 genes with two CDs, only one, in this case, the N-terminal CD, has deaminase activity (58). mA3^BALB^ was also identified in *M. spretus*, a sister species that is sympatric with and partially interfertile with *Mus musculus domesticus* (59). Our previous work found that mA3^BALB^ (defined by the absence of the MLV LTR and presence of exon 5) predominates in western Europe (60), and here, we also found it throughout eastern Asia based on sequence and lack of the MLV LTR (Fig. 4B). In contrast to this wide distribution of mA3^BALB^, mA3^B6^ was found in only two mice, *M. m. domesticus*-WSB and -Lewes, wild-derived strains originating in Maryland and Delaware, but these mice may have been subject to the reported inadvertent cross-contamination of such wild-derived strains with laboratory strains (57). The 18 novel mA3 variants were widely distributed from central Europe to the Pacific with geographic concentrations of specific variants such as mA3–8 and mA3–15 (Fig. 4B).

The 18 novel mA3 haplotypes in *M. musculus* include substitutions at 10 sites previously shown to be under positive selection in the broader set of species within the *Mus* genus (13). Therefore, we assessed the *Mus musculus* mA3 genes for signatures of positive selection, identified by the ratio of nonsynonymous mutations relative to synonymous mutations. Such an imbalance is characteristic of genes under selective pressure, particularly genes that function in defense, and can flag residues likely to have important functional roles.

**Fig 5:**
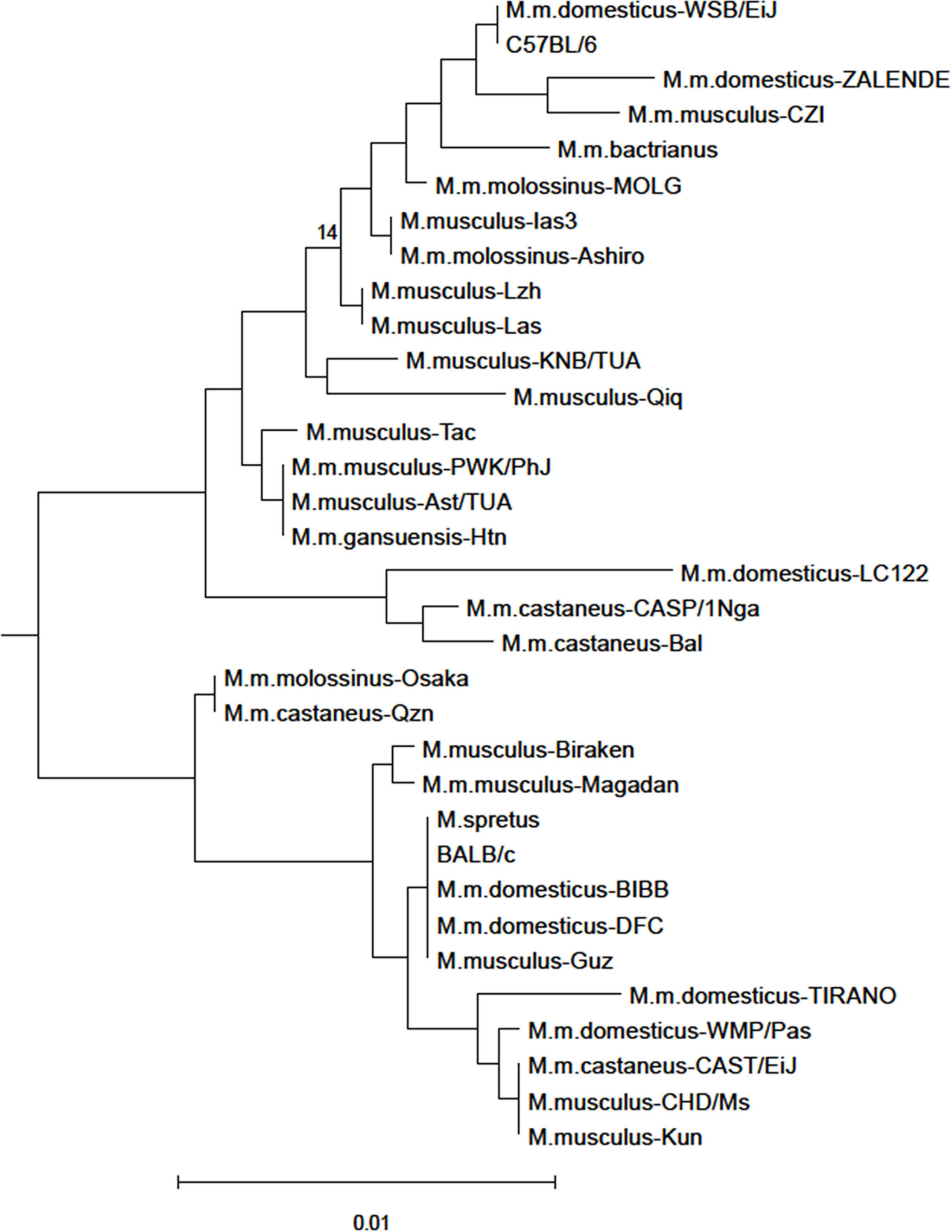
Phylogenetic tree of mA3 sequence variants used for selection analysis. Exons 2–4 and 6–8 of mA3 from the indicated species were aligned and a maximum-likelihood tree was generated using RaxML with 500 bootstraps. The tree was rooted using the mA3 sequence of *Mus dunni* (GenBank accession number GQ871506.1). Bootstrap values that are less than 70 are shown at the relevant nodes. Scale bar represents 0.01 nucleotide substitutions per site.

The *M. musculus* mA3 phylogenetic tree generated for this analysis identifies two clades broadly corresponding to the two inbred strain alleles (Fig. 5). Twelve mA3 sites were found to be under positive selection, two of which were identified by all three programs used (PAML, MEME, FEL) (Table 5). Six of the seven positively selected sites in the active CD (34G, 37K, 38G, 134V, 135Q, 136D) had also been shown to be under positive selection in *Mus* (13), as had the three adjacent sites near the N-terminal end of the inactive CD (200M, 201D, 202P), and two additional sites in this CD (225R, 237T). These data indicate that mA3 has been engaged in genetic conflicts in *M. musculus* subspecies and identify multiple sites that likely have functional significance.

**TABLE 5:** Positive selection of sites in mA3 from subspecies of *M. musculus*.

Gene	Codon frequency	ω^0^^*a*^	M7–M8		Tree length^*c*^	*dN/dS* (%)	Residues with dN/dS of >1 and pr of >0.95^*d*^
			2δ^*b*^	*P*-value			
*Apobec3*	f3 × 4	0.3	46.104	0.000000000	0.19702	27.444 (5.95)	**34G**, **37K,^*e*^** 38G, 134V, **135Q**, **136D**, **184T**, 200M, **201D**, **202P,^*e*^** 225R, 273T
	f3 × 4	1.7	46.104	0.000000000	0.19702	27.444 (5.95)	**34G,**^*a*^ **37K,^*e*^** 38G, 134V, **135Q**, **136D**, **184T**, 200M, **201D**, **202P,^*e*^** 225R, 273T

^*a*^ ω^o^ denotes the initial seed value of ω (ratio of nonsynonymous to synonymous substitution rates).

^*b*^ 2δ, two times the difference of the natural log values of the maximum likelihood from pairwise comparisons of the different models.

^*c*^ Tree length is defined as the sum of the nucleotide substitutions per codon at each branch.

^*d*^ Residue numbers are based on the mA3^B6^ sequence in GenBank accession number NM_030255 (https://www.ncbi.nlm.nih.gov/nuccore/NM_030255.3/). pr, posterior probability. Boldface type represents pr > 0.99.

^*e*^ Residues found to be under positive selection by the FEL and MEME programs of the Datamonkey web server (61); all listed residues were identified by PAML4.9 (62).

To ascertain if specific mA3 variations affect the hypermutation profiles of *Mtvs* carried by these wild mice, we cloned and sequenced *Mtv env, sag,* and *pol* genes from wild mice carrying 12 of the 20 mA3 variants (Table S2). In contrast to the *Mtvs* from sequenced genomes, the majority of these sequences were, at best, lightly edited, which is generally consistent with the low levels observed in newly acquired MMTV proviruses (14, 33, 63).

These results describe a remarkable level of evolutionary diversification within a single widely dispersed species that originated only 0.5 MYA and furthermore show that sites that distinguish the two functionally different inbred strain mA3 alleles display extensive polymorphisms in wild mice and strong positive selection, which is evidence of exposure to high-impact genetic conflicts.

## DISCUSSION

These data show that the deaminase function of mA3 has significantly altered the integrity and functionality of *Mtvs*, and that mA3 has rapidly evolved in *M. musculus,* generating at least 20 variants in house mice, only two of which were captured by the classical inbred strains. Although previous reports suggest that MMTVs are not particularly sensitive to mA3 deamination (14, 33, 63), here, we found examples of very high levels of hypermutation. Our results also offer insights into the process of mA3 deamination of retroviral DNA: mA3 can introduce mutations uniquely into the nascent proviral 3′ LTR, generate *Mtv* proviruses displaying the target site preferences of the two laboratory mouse mA3 alleles (33), and create new preferred deamination target sites as it modifies adjacent cytosines. These observations underscore the involvement of mA3 deamination as a contributor to defense against MMTVs.

The 18 haplotype variants of mA3 in *Mus musculus* subspecies are distinguished by different combinations of replacement mutations involving the 15 sites that distinguish the two laboratory mouse variants (7). Ten of these 15 sites showed significant levels of positive selection in our previous analysis of mA3 in the genus *Mus* (13). In homology models, six sites in the N-terminal active CD form two clusters (34G, 37K, 38G and 134V, 135Q, 136D). The predicted structures suggested sequence variants in these clusters could modify electrostatic interactions and positioning, and our cluster exchange experiments identified differences in antiviral activity against Friend MLV (13). These two clusters face each other across the substrate groove (13)**,** one of which aligns with a loop in hA3G. Loop grafting experiments involving hAID, hA3F, and hA3G showed small differences in enzymatic activity but more significant effects on target specificity (64–67). This suggests a possible role in substrate selection for the similarly situated mouse determinants. None of the other identified G > A mismatches modify the two HAE active deamination motifs or the PCX_2_C domains that coordinate zinc ions. There are also five sites under positive selection in the C-terminal CD, one that was identified with all three programs with high significance, suggesting these sites also have functional importance. The inactive CD of hA3G has roles in packaging, dimerization, and interactions with the HIV-1 Vif (68), and while the inactive mA3 CD has also been shown to govern encapsidation, residues linked to this function do not overlap the three positively selected mA3 residues at the N-terminal end of this CD (69, 70).

Both inbred strain mA3 alleles are known to have antiviral activity. mA3 has two antiretroviral activities: mutagenesis by deamination and inhibition of reverse transcriptase (RT), and the mA3^BALB^ and mA3^B6^ proteins show different activity levels in both functions (33, 70). Direct measurements show that mA3^BALB^ has somewhat higher deaminase activity than mA3^B6^, whereas inhibition of RT has been shown to be more substantial for mA3^B6^ than mA3^BALB^ (33, 71–73), which may account for the stronger inhibition of retrovirus replication and pathogenesis by mA3^B6^ (7, 10). Although retroviruses that naturally infect mice, like MMTVs and MLVs, are subject to mA3 restriction, previous studies reported generally low levels of mA3 deamination in infected mice or cells (14, 17, 19, 71), which led to the conclusion that deamination is not critical for restriction (33, 70). However, our data show that mA3 mutagenesis can be significant and functionally consequential. The fact that MMTV proviruses preserved as *Mtvs* have sustained greater levels of hypermutation than newly acquired proviruses may simply reflect the fact that such highly disabled *Mtvs* are either bypassed for removal by natural selection pressures or have retained or acquired beneficial functions, like *sag* expression that inhibits MMTV infection.

Our observations contrast with previous reports of the generally low level of mA3 hypermutation of mouse retroviruses, but multiple factors can influence mA3 hypermutation, such as variability in the extent of mA3 packaging (33, 74). More recent studies identified Rem as an antagonist of AID-induced hypermutation (75), linked the variability of hypermutation in BALB and B6 mice to *rem* expression, and suggested that Rem can affect mA3 (76). Also, the cellular gene DHX15 was recently identified as an mA3 antagonist (63). DHX15 can be packaged in MLV virions and binds to the region encoding the active deaminase site in the active CD. Although this 20 amino acid binding region shows no sequence variability in the 20 mA3 variants, sequence polymorphisms in DHX15 that affect binding, expression levels, or packaging efficiency could differentially affect hypermutation levels.

Once integrated, proviruses become cellular sequences and are subject to cellular mutational processes, but the observed *Mtv* hypermutation profiles are generally consistent with mA3 editing of proviral DNA prior to germline integration rather than the accumulation of such mutations over long evolutionary time frames. Somatic hypermutation has been well documented. AID-induced hypermutation is critical to the development of diversified antibodies in B cells, and somatic hypermutations have been linked to chromosomal translocations and single nucleotide mutations associated with various human malignancies (77). However, germline hypermutation is very rare, and the few observed cases are largely attributable to mutations in paternal DNA repair genes (78), enzymes also known to repair proviral DNA by removing uracils (79). Over evolutionary time, rare, sequentially acquired mA3-generated mutations could potentially augment the hypermutation profile of specific *Mtvs*, but the strand specificity of the extreme excess of G > A vs C > T mutations in these *Mtvs* is consistent with mA3 activity prior to endogenization. Furthermore, the G > A mutations are restricted to the proviruses and do not extend into flanking cellular DNA, as shown for *Mtv21* (Data Files S1 and S2), arguing against regional germline mutation. Finally, while it is also possible for mA3 edits to accumulate in ERV genomes that undergo sequential rounds of retrotransposition and reintegration with exposure to mA3, retrotransposition is rare for most ERV groups, and we found only one example among the *Mtvs* in the relationship between *Mtv17* and *Mtv23*, an example which did not involve additional hypermutation.

The APOBECs can serve as drivers of diversification, altering proviruses from potentially dangerous pathogenic agents to benign sequences containing protein coding stretches and regulatory elements, features ready to be appropriated to serve host functions (80). Among the coding genes, 12 of 14 2-LTR *M. musculus Mtvs* have lost *env*, as also well documented for most human ERV groups (for example, HERV-W [81]). While a functional Env can be dangerous to the host because of its immunogenic and fusogenic properties, interestingly, only 5 of these 14 *Mtvs* have lost the *env*-derived *rem* gene ORF, suggesting its retention may be neutral or even beneficial. Also notably, all but two *Mtvs* have retained an intact *sag*, a gene that, when endogenized, protects against exogenous infection, like other co-opted retroviral genes with antiviral functions, such as *Fv1*, *Fv4,* and *Rmcf* (1). The *Mtvs* also retain regulatory sequences including transcription factor binding sites, some of which remain intact and ready to be repurposed.

The rapid evolution of mA3 in wild mouse populations may reflect the pathogenic threats these mice have encountered. House mice acquired *Mtvs* and MLV ERVs at about the same time, just prior to *M. musculus* subspeciation, ~0.5 MYA, but, unlike MMTVs, the MLVs encode two factors that antagonize mA3, p50 and glyco-Gag (30, 82). Cleavage by the MLV protease may also inactivate mA3 (55), and the unusually rapid rate of processivity of the MMTV polymerase can reduce the level of A3 mutagenesis (83). Glyco-Gag is present in some but not all MLV subgroups (60). The polytropic MLV ERV subtypes (*Mpmvs* and *Pmvs*), which lack glyco-Gag, have been subject to mA3 mutagenesis (19), and the geographic distribution of mice with these ERVs (*Mpmvs* and *Pmvs*) roughly coincides with the distribution of mA3^BALB^ in Europe (53). Conversely, MLV subtypes encoding the protective glyco-Gag (most *Xmvs* and *Emvs*) are found in wild mice from central Europe through Asia (53, 60). The observed mA3 diversification may thus have been driven by an arms race with the more mA3-resistant MLVs in these Asian mice, an arms race which has also resulted in glyco-Gag diversification. The presence of glyco-Gag is not sufficient for protection; the extra glycosylation site in the MoMLV glyco-Gag correlates with its decreased susceptibility to mA3 hypermutation compared to AKV MLV (84). The MMTVs do not have a sequence comparable to glyco-Gag, but while the observed resistance of MMTVs to mA3 deamination has not been explained by a virus-encoded antagonist, Rem has been implicated in the antagonism of another family member, AID (75), and may influence mA3 hypermutation in some mouse strains (76).

mA3^BALB^ is carried by *M. spretus*, a species sympatric with and partially interfertile with *M. m. domesticus*. There is documented introgression between these taxa that tends to be unidirectional (85), and we previously showed that *M. spretus* has also acquired the polytropic MLV subtype from *M. m. domesticus,* which, along with the *Mtvs,* are otherwise restricted to *M. musculus* (32, 86). Introgression from *domesticus* to *M. spretus* has resulted in the transfer of multiple cellular genes, including one that serves a clear adaptive role, *Vkorc1,* which is responsible for resistance to warfarin, a rat poison (87). Among the other genes transferred and fixed in *M. spretus* is mA3 (85), shown here to be mA3^BALB^, another example of a beneficial gene introgressed from *M. musculus* to its sister species, a species needing protection from the potentially pathogenic and mutagenic MLVs and MMTVs it has also acquired from *M. m. domesticus*.

We also show here, for the first time, that mA3 substitutions can differentially mutate 3′ proviral LTRs. During the late stages of reverse transcription, the minus strand of the nascent 3′ LTR is displaced, becoming transiently single-stranded for the second time while the 5′ LTR is completed (88, 89); this would make the 3′ LTR subject to further mutagenesis by mA3. While G > A mismatches unique to 3′ LTRs have not been reported for other proviruses, we detected this phenomenon among the MLVs; other well-studied ERVs are considerably older, and their accumulated mutations could easily obscure a few G > A differences. Acquisition of these 3′ LTR mutations in multiple MMTVs may be due to their unusually large LTRs (>1,300 bp), presenting a larger target than the LTRs of other retroviruses. Other explanations, like ectopic recombination, accumulation of mutations subsequent to proviral insertion, additional rounds of editing in retrotransposed proviruses, or correction by base excision repair (39, 45, 79), cannot account for the 3′ LTR specific pattern of G > A mismatches observed here. Similarly, the presence of identical LTRs in *Mtv23*, unlike its *Mtv17* progenitor, likely resulted when an *Mtv17* transcript was packaged and transmitted to a new cell, followed by reverse transcription in which the *Mtv17* U3 with the extra G > A mutations served as template the proviral LTRs in the resulting *Mtv23* provirus.

The observed differences in proviral LTRs have implications, not only for how mA3 operates during reverse transcription, but for the application of a widely used method to determine the age of ERVs. Over time, ERVs accumulate mutations, insertions, and deletions. Because these events are cumulative at a defined rate, they can be used to estimate age from integration by comparing the LTRs of individual ERVs (90, 91). Such age estimates would be distorted if mA3 hypermutation introduces multiple mutations in one LTR just prior to integration. Also, viral genome-wide hypermutations can distort age estimates that are based on the phylogenetic trees of related ERVs, resulting in inaccurately long branch lengths and clustering of otherwise more distantly related ERVs due to shared mutations, as we observed for the extensively hypermutated ERVs *Mtv21* and *61*. Such timeline distortions resulting from G > A mutational load have also been described for other ERVs (for example, see references 19, 54, 80).

Finally, it has long been recognized that A3 mutations accumulate in C tracts (36, 37), producing runs of G > A substitutions in the proviral plus strand. Our analysis of such sites in the highly mutated *Mtv21* suggests that concurrent mA3 mutagenesis can produce new target sites for deamination in C tracts that would not otherwise offer preferred target sites. Such newly acquired preferred targets also increase the overall level of mutagenesis as reported by a study on the time course of HIV-1 deamination by hA3G, which found that while 3.5% of HIV-1 Gs are mutated after 4 hours, this increases to 10% at 24 hours with an associated reduction in target site specificity. This differential in the extent of mutagenesis and apparent change in target site specificity defined by the reference sequence is explainable by the generation of new target sites by hypermutation during the initial phases of mutagenesis (92).

## MATERIALS AND METHODS

### Sources of mouse DNAs

Wild mouse DNAs were isolated from mice maintained in our laboratory or obtained from colonies maintained by M. Potter (NCI, Bethesda, MD) or trapped by S. Rasheed (University of Southern California, Los Angeles). Additional DNAs from wild-caught or wild-derived mice were purchased from The Jackson Laboratory (Bar Harbor, ME) and from the RIKEN BioResource Center (Tsukuba, Japan) (Table S6). DNAs from classical inbred strains listed in Table S5 were purchased or isolated from mice maintained in our laboratory or obtained from The Jackson Laboratory or RIKEN.

### *Mtv* and *Apobec3* sequences

Seventeen sequenced mouse genomes (93) were screened for MMTV-related sequences by BLAST (blast.ncbi.nlm.nih.gov) (94) using as probe *Mtv1* and infectious MMTVs (GenBank accession numbers AF228550 and AF033807). Twenty-nine *Mtvs* were identified in 12 classical inbred strains and four wild-derived strains developed from PWK/PhJ (*M. m. musculus*), CAST/EiJ (*M. m. castaneus*), WSB/EiJ (*M. m. domesticus*), and SPRET/EiJ (*M. spretus*) as described previously (32). Full-length *Apobec3* genes from these sequenced genomes were obtained from Ensembl, release 113 (95). Other previously sequenced mA3 genes were reported in reference 13 and for mA3^BALB^ (GenBank accession number BC003314).

Additional MMTV-related DNA segments for hypermutation analysis were amplified by PCR from DNAs extracted from wild-derived inbred strains and wild-caught mice using primers designed to amplify *env, pol,* and *sag* sequences (Table S7). Partial mA3 sequences were amplified from mouse genomic DNAs using primers designed from mA3 coding, flanking, or intron sequences based on the mouse C57BL/6J reference genome (GenBank accession number NC_000081.7) (Table S7). PCRs were performed using AmpliTaq Gold (Thermo Fisher, Waltham, MA) with the following program: 95°C for 7 min; 35 cycles of 94°C for 60 s, 58.6°C for 60 s, and 72°C for 60 s for target sequences up to 1 kb and 180 s for targets of 2 kb–3 kb; 72°C for 7 min; and 12°C hold. In some cases, PCRs used a blend of Hot Start Taq and Deep Vent DNA Polymerase (New England Biolabs, Ipswich, MA) with the program 94°C hot start; 35 cycles of 94°C for 30 s, 60°C for 60 s, 65°C for 120 s; 65°C for 10 min; and 12°C hold.

The hypermutation analysis of *Mtv* sequences included previously sequenced *Mtv sag* genes (GenBank accession numbers PQ434792-PQ434820), *env* genes (PQ434833-PQ434848 and PQ434850-PQ434857) (32), and newly sequenced *pol* genes (GenBank accession numbers PV831901-PV831908). *M. musculus*-derived *Mtv* sequences were assessed for mismatches using alignments with *Mtv1,* which was the best sequence match among the previously sequenced full-length *Mtvs* and MMTV genomes. The *M. spretus Mtvs* were aligned with a consensus derived from the full-length *M. spretus Mtv32, 34,* and *36–38*. Newly sequenced partial mA3 sequences were deposited under GenBank accession numbers PV831856-PV831900. Alignments were created using MUSCLE or ClustalW from EMBL-EBI (96).

Amplified mA3 and *Mtv* DNAs were cloned into the PCR2.1 TOPO1 plasmid (Thermo Fisher), and three to nine clones of each were sequenced in-house or by Psomagen (Rockville, MD).

### Phylogenetic analysis

mA3 exons 2–4 and 6–8 were amplified from 50 wild mouse genomic DNAs and nearly full-length cDNAs were produced from RNA isolated from two mice. Sequences were aligned using ClustalW as implemented in Geneious Prime 2022.1.1 using the default settings. Maximum-likelihood phylogenetic trees were generated using the RaxML program with the General Time Reversible + G + I model and 500 bootstraps for branch support (97).

Sliding window analysis of substitutions in various *Mtvs* was done by first manually assigning a number to each nucleotide based on a comparison to *Mtv1* (0 for no change, 1 for G > A substitutions, 2 for A > G substitutions, and 3 for C > T substitutions). These numbers were then plotted in Microsoft Excel using the COUNTIF and OFFSET functions to establish a sliding window of 400 bases moving at a 100 step size.

### Maximum-likelihood test for detecting positive selection

To test for codon evolution in mA3 sequences, we used the codeml program of PAML 4.9 (62) as well as two programs on the DataMonkey web server: FEL and MEME (61). Initial positive-selection analyses were performed using the MEME and REL programs from DataMonkey web server with recommended settings and *P* < 0.05. For the analysis using PAML 4.9, likelihood ratio tests were performed to compare M8, a positive-selection model with beta distribution of *d*N/*d*S values, to M7, a neutral model with beta distribution that does not allow positive selection. Chi-square analysis was done, and the model that fit the data better was selected using a *P*-value of 0.01. The F3 × 4 codon frequency model in codeml of PAML 4.9 was used to determine the positively selected residues in mA3, with two separate initial seed values of ω: 0.3 and 1.7. Posterior probabilities of codons under positive selection were inferred using the NEB algorithm in the M8 model (Table 5).

## Data Availability

Sequence data were deposited to GenBank for partial sequences of wild mouse mA3 genes under accession numbers PV831856-PV831900 and for *Mtv pol* genes under PV831901-PV831908. All other data are available without restriction upon written request to the corresponding author.

## SUPPLEMENTAL MATERIAL

Supplemental material jvi.01251-25-s0001.pdf Tables S1 to S7, Figures S1 to S3, and Data Files S1 and S2.

ASM does not own the copyrights to Supplemental Material that may be linked to, or accessed through, an article. The authors have granted ASM a non-exclusive, world-wide license to publish the Supplemental Material files. Please contact the corresponding author directly for reuse.
