# Supplementary material for: Apobec3 shows rapid evolution in house mouse subspecies and unusual hypermutation patterns of endogenous mouse mammary tumor viruses
Source: J Virol. 2025 Sep 16;99(10):e01251-25. doi: 10.1128/jvi.01251-25 (PMC12548423; doi:10.1128/jvi.01251-25)
Supplement: Supplemental material — Tables S1 to S7, Figures S1 to S3, and Data Files S1 and S2. [file jvi.01251-25-s0001.pdf]

Supplementary Files for

Apobec3 shows rapid evolution in house mouse subspecies and produces unusual hypermutation patterns in endogenous mouse mammary tumor viruses

Esther Shaffer, Guney Boso, Reza Sadjadpour, Venkat V.S.R.K. Yedavalli, Oscar Lam and Christine A. Kozak

Supplementary Tables:

Table S1. Mutation profiles of solo LTR *Mtvs* extracted from sequenced mouse genomes. *Mus spretus Mtvs* (*Mtv58-C0*) were compared with a *spretus Mtv* consensus and the *M.musculus Mtvs* with *Mtv1*.

Table S2. Mutation profiles of *Mtv env, pol* and *sag* segments cloned from wild-derived or wild-caught *Mus musculus*.

Table S3. Open reading frames in coding genes of full-length *Mtvs* in the sequenced *Mus musculus* and *Mus spretus* genomes.

Table S4. Amino acid substitutions and stop codons due to G>A mismatches in coding regions of four hypermutated *Mtvs*.

Table S5. Distribution of mA3 alleles in classical inbred strains of laboratory mice.

Table S6. List of wild mice.

Table S7. Primers used for PCR to amplify segments of mA3 and MMTV related sequences.

Supplementary Figures:

Figure S1. Comparative analysis of the 5' and 3' LTRs of *Mpmv5, Pmv23, Mpmv7* and *Mpmv4C* for shared or 3'LTR-specific G>A mismatches.

Figure S2. Sequence contexts of all mA3 hypermutations at the -1 and -2 nucleotide positions relative to minus strand targeted cytosines in individual *Mtvs*: **A)** *Mtvs* with moderate levels of hypermutation showing mA3^{B6}-like target preferences; **B)** heavily mutated *Mtv21* and *-C1* which show mA3^{BALB}-like target preferences; **C)** 5' and 3' halves of *MtvC1*; **D)** shared G>A mutations in *Mtv* sets with different sequence contexts; **E)** the subset of *Mtv21* mutations with no other mutations within 3 nucleotide positions. Red bolding marks the most frequently used sequence context.

Figure S3. Sequence variants of mA3 exons 2-4 in wild mice. At the top is a diagram of the first four mA3 exons showing the locations of sites that distinguish mA3^{BALB} and mA3^{B6}. The chart shows amino acid residues at these and two additional sites for 19 wild mice.

Supplementary Datafiles:

Datafile S1. Alignment of *Mtv21* with *Mtv1* showing mismatched nucleotides, with G>A mismatches in red.

Datafile S2. Sequence mismatches in the genomic sequences flanking *Mtv21* in the NZO/HILtJ genome relative to the orthologous segment in the B6 reference genome.

Table S1. Mutation profiles of solo LTR *Mtvs* extracted from sequenced mouse genomes. *Mus spretus Mtvs* (*Mtv58-60*) were compared with a *spretus Mtv* consensus and the *M.musculus Mtvs* with *Mtv1*.

Mtv	**Mismatches**				**%G>A/Total**
	**G>A**	**C>T**	**A>G**	**Total**	
Mtv14	12	8	5	42	28
Mtv58	17	20	17	94	18
Mtv59	16	21	16	93	17
Mtv60	9	19	15	85	11
Mtv63	9	13	8	52	17
Mtv64	8	8	6	40	20

Table S2. Mutation profiles of *Mtv env, pol* and *sag* segments cloned from wild-derived or wild-caught *M. musculus*.

Gene (size, kb)	Mouse[a]	Apobec3 Type[b]	Mismatches					Runs of G>A			Internal Stop Codons
			G>A	A>G	C>T	Total	%G>A/total	2	3	4	
env (2.1)	*M.m.castaneus*-Bal	12	18	12	12	61	29.5				
	M.m.castaneus-Qzn	17	17	10	13	60	28.3				
	M.m.domesticus-ABUR	mA3^{BALB}	18	12	18	74	24.3				
	M.m.domesticus-LC122[a]	14	24	6	4	38	63.2	2			
	M.m.musculus-Akt	mA3^{BALB}	45	24	25	146	30.8				2
	M.m.musculus-Birakan[a]	18	55	15	18	121	45.5	1	1		5
	M.m.musculus-CZI	2	26	14	31	73	35.6				
	M.m.musculus-Krk1	mA3^{BALB}	22	12	16	76	28.9				
	M.m.musculus-Skive	mA3^{BALB}	45	25	25	150	30.0				2
	M.m.musculus-MYL	8	20	15	20	84	23.8				
env (1.0)	*M.m.domesticus*-JJD	mA3^{BALB}	34	25	18	128	26.6				
	M.m.musculus-Donetsk	mA3^{BALB}	9	10	6	35	25.7				
	M.m.musculus-Moscow	mA3^{BALB}	10	9	6	35	28.6				
	M.m.musculus-Novii	15	11	9	6	36	30.6				
sag (0.9)	*M.m.castaneus*-Bal	12	10	4	7	35	28.6	1			
	M.m.castaneus-Qzn1	17	11	2	8	33	33.3				
	M.m.castaneus-Qzn2	17	8	2	9	32	25.0	1			
	M.m.domesticus-LC122-1	14	9	0	5	23	39.1				
	M.m.domesticus-LC122-2	14	8	5	4	27	29.6				
	M.m.domesticus-LC122-3[a]	14	17	6	4	35	48.6				
	M.m.domesticus-LEWES1	mA3^{B6}	11	3	6	33	33.3				
	M.m.domesticus-LEWES2	mA3^{B6}	8	0	5	22	36.3				
	M.m.domesticus-LEWES3	mA3^{B6}	18	11	6	40	45.0				
	M.m.domesticus-ZALENDE	3	7	2	9	28	25.0				
	M.m.musculus-Akt1	mA3^{BALB}	7	7	7	39	17.9	1			
	M.m.musculus-Akt2	mA3^{BALB}	7	11	12	51	13.7	1			
	M.m.musculus-Ast	8	23	6	18	80	28.8	1			
	M.m.musculus-CZ1	2	18	11	13	52	34.6				
	M.m.musculus-Krk	mA3^{BALB}	8	6	11	39	20.5				
	M.m.musculus-Krk2	mA3^{BALB}	17	6	16	66	25.8	1			
	M.m.musculus-Krk3	mA3^{BALB}	7	6	10	38	18.4	1			
	M.m.musculus-Magadan1	18	10	8	9	40	25.0				
	M.m.musculus-Magadan2	18	11	10	15	46	23.9				
	M.m.musculus-Novii	15	12	11	17	73	16.4	1			
	M.m.musculus-Skive2	mA3^{BALB}	11	2	6	32	34.4	1			
	M.m.musculus-Skive3	mA3^{BALB}	10	2	5	25	40.0	1			
	M.m.musculus-Skive4	mA3^{BALB}	23	6	18	79	29.1				
	M.m.domesticus-TIRANO	16	11	2	7	33	33.3				
	M.m.musculus-VEJ1	mA3^{BALB}	12	2	6	33	36.4	1			
	M.m.musculus-VEJ2	mA3^{BALB}	9	0	5	24	37.5				
	M.m.musculus-VEJ3[a]	mA3^{BALB}	19	6	4	42	45.2				
pol (2.1)	*M.m.castaneus*-Qzn	17	17	7	20	65	26.2				
	M.m.musculus-Novii-1	15	21	16	19	90	23.3	1			
	M.m.musculus-Novii-2	15	25	36	31	157	15.9				
	M.m.musculus-Novii-3[a]	15	76	13	18	140	54.3	7	2		
	M.m.musculus-Magadan-1	18	21	24	16	96	21.9	1			
	M.m.musculus-Magadan-2[a]	18	42	14	17	107	39.3	3			
	M.m.musculus-Magadan-3	18	32	17	18	99	32.3	2			
pol (1.1)	*M.m.molossinus*-MOLG	5	12	6	15	50	24.0				

[a]G>A mismatches > twice those for A>G and C>T.
[b]mA3 type from Figure 4A.

Table S3. Open reading frames in coding genes of full length *Mtvs* in the sequenced *Mus musculus* and *Mus spretus* genomes.

Chr	*Mtv*	Strain[a]	ORFs[b]					
			gag	*pro*	*pol*	*env*	*rem*	*sag*
1	7	DBA/2J	+	+	+	-	-	+
3	55	LP/J	+	+	+	-	+	+
4	17	C57BL/6J	+	+	-	-	-	+
4	13	FVB/NJ	+	+	-	+	+	+
6	23	AKR/J	+	+	-	-	-	+
6	8	C57BL/6J	+	+	+	-	+	+
7	1	C3H/HeJ	+	+	+	+	+	+
8	21	NZOHILtJ	-	-	-	-	-	-
11	3	NOD/HILtJ	+	+	-	-	+	+
12	9	C57BL/6J	+	+	+	-	-	+
14	11	C3H/HeJ	+	+	-	-	+	+
15	57	LP/J	-	+	-	-	+	+
5	32	*M.spretus*	+	+	+	+	+	+
6	33	*M.spretus*	+	+	+	+	+	+
6	34	*M.spretus*	+	+	+	+	+	+
15	36	*M.spretus*	+	+	+	+	+	+
16	37	*M.spretus*	+	+	-	+	+	+
18	38	*M.spretus*	+	+	+	+	+	+
X	61	CAST/EiJ	+	+	-	-	+	-
11	62	PWK/PhJ	-	+	-	-	+	+

[a]One strain is listed for each *Mtv* carried by multiple strains.

[b]+, ORF present; -, ORF absent due to stop codons at Trp codons.

Table S4. Amino acid substitutions and stop codons due to G>A mismatches in coding regions of four hypermutated *Mtvs*.

Mtv	Gene[1]		Codons with G>A mutations		
			Total	Amino Acid Substitutions	Premature Termination Codons
MtvC1	*gag*		48	16	
	pro		21	6	
	pol		80	43	3
	env	*env*	129	89	4
		env/*rem*-SP	21	11	
		rem-CT	31	18	
	sag[2]		70	56	
Mtv57	*gag*		33	18	4
	pro		25	13	
	pol		41	28	
	env	*env*	38	18	3
		env/*rem*-SP	6	2	
		rem-CT	10	6	
	sag		19	14	
Mtv17	*gag*		10	5	
	pro		6	5	
	pol		32	16	4
	env	*env*	27	12	3
		env/*rem*-SP	5	1	1
		rem-CT	5	4	
	sag		9	9	
MtvC2	*gag*		10	6	1
	pro		11	8	
	pol		30	17	3
	env	*env*	32	24	1
		env/*rem*-SP	5	4	
		rem-CT	9	5	
	sag		9	7	

[1]*env* data are provided for full length *env* and separately for two cleavage products, the *env*/*rem* signal peptide (SP) and the *rem* C-terminal fragment (CT).

[2]Corrected for an internal 2-nucleotide frameshifting deletion.

Table S5. Distribution of mA3 alleles in classical inbred strains of laboratory mice.

Inbred Strains		Typing Methods[a]
$mA3^{B6}$	$mA3^{BALB}$	
C57BL/6J,NZO/HILtJ	129X1/SvJ, A/J, AKR/J, BALB/cJ, C3H/HeJ, CBA/J, DBA/2J, FVB/NJ, LP/J, NOD/ShiLtJ	Sequenced genomes
NZB/BINJ	A.By, B10.BR, C57BR/cdJ, C57L/J, MA/MyJ	Sequence polymorphisms, MLV LTR, Exon5 determinants
RF/J	C58/J, NFS/N, NZM2410/J, NZO, NZW/LacJ, SEC/1ReJ	Sequence polymorphisms, MLV LTR
	I/LnJ, SWR/J, YBR/EiJ	MLV LTR, Exon5 determinants
	A/WySn,B10.A, SB/LeJ	Exon5 determinants
NZL/LtJ, RIIIS/J	CBA/CaJ, F/St, GRS, KK/HIJ, LG/J, NON/ShiLtJ, NOR/LtJ, P/J, PL/J, SEA/GnJ, SEC/1ReJ, SJL/J, SM/J, TALLYHO/JngJ,	MLV LTR

[a]mA3 sequences were obtained from sequenced genomes, mRNA or PCR-derived exon-intron segments; the presence or absence of the MLV LTR by sequence of the intron downstream of Exon 2 or by PCR, and Exon 5 determinants were identified in that exon or its upstream intron.

Table S6. List of wild mice.

Mus musculus subspecies[1]	Designation	Trapping location	DNA Code	Source	Used to sequence	
					mA3 (exons)	*Mtv genes*
bactrianus	Bac	Mashhad, Iran	MG-0417	RIKEN	(2-4,6-8)	
castaneus	Bal	Bandar, Sumatra, Indonesia	MG-5121	RIKEN	(2-4,6-8)	*env,sag*
	CASP/1Nga	Los Banos Philippines		RIKEN	(2-4,6-8)	
	Qzn	Quezon City, Philippines	MG-0421	RIKEN	(2-4,6-8)	*pol,sag*
domesticus	ABUR	Abu Rawash, Egypt		Potter	(2-4,6-8)	*env*
	BIBB	Sede Boger/1, Israel	MG-0377	RIKEN	(2-4)	
	DFC	Corse, France	MG-0385	RIKEN	(2-4,6-8)	
	JJD, J.J.Downs	Ridgely, MD		Potter		*env*
	LC122	Lake Casitas, CA		Rasheed	(2-4,6-8)	*sag*
	Lewes	Lewes, DE		Potter	(2-4)	*sag*
	PERC/EiJ	Rimac Valley, Peru		Jackson	(2-4)	
	SK/Cam	Skokholm Is., U.K.	MG-0067	RIKEN	(2-4)	
	TIRANO, Posch-1	Tirano, Italy		Potter	(2-4,6-8)	*sag*
	ZALENDE, Posch-2	Zalende, Switzerland		Potter		*pol,sag*
	WMP/PasDnJ	Monastir, Tunisia		RIKEN	(2-4,6-8)	
molossinus	MOLG	Fukuoka, Kyushu, Japan		Potter	(2-4,6-8)	
	Ashiro	Ashiro, Iwate, Japan	MG-0257	RIKEN	(2-4,6-8)	
	Osaka	Takatsuki, Osaka, Japan	MG-0240	RIKEN	(2-4,6-8)	
musculus	Akt/TUA	Aktubinsh, Kazakhstan		RIKEN	(2-4)	*env,sag*
	BLG2/Ms	Toshevo, Bulgaria		RIKEN	(2-4)	
	CZI, CZECHI	Morovia, Czech Republic		Potter		*env,sag*
	Donetsk	Donetsk, Ukraine	MG-3065	RIKEN	(2-4)	*env*
	Krk1	Krakow, Poland	MG-5235	RIKEN	(2-4)	*env,sag*
	Magadan	Magadan, Russia	MG-3063	RIKEN	(2-4,6-8)	*pol,sag*
	MYL	Ljubljana Slovenia, (Yugoslavia)	MG-0399	RIKEN	(2-4)	*env*
	Skive	Skive, Denmark		Potter	(2-4)	*env,sag*
	VEJ	Vejrumbro, Denmark		Potter	(2-4)	*sag*
musculus (gansuensis)	CHD/Ms (*M.m.gansuensis*)	Chengdu, China			(2-4,6-8)	
	Htn	Hotan, China	MG-0762	RIKEN	(2-4,6-8)	
musculus (tantillus)	Las	Lasa, China	MG-0723	RIKEN	(2-4,6-8)	
musculus (wagneri)	Ast/TUA	Astrakhan, Russia		RIKEN	(2-4,6-8)	*sag*
musculus (wagneri)	KNB/TUA	Balkash Lake, Kazakhstan		RIKEN	(2-4,6-8)	
spp.	Birakan	Birakan settlement, Khabarovski region, Western Birodidzan city, U.S.S.R.	MG-3127	RIKEN	(2-4,6-8)	*env*
	Dal	Dali, China	MG-0788	RIKEN	(2-4)	
	Grozny	North Caucasus, Grozny City, Russia	MG-3010	RIKEN	(2-4)	
	Gui	Guilin, China	MG-0502	RIKEN	(2-4)	
	Guz(Shi)	Guangzhou , China	MG-0503	RIKEN	(2-4,6-8)	
	Ias3	Suweon, Korea	MG-5018	RIKEN	(2-4,6-8)	
	Jin	Jiangyin, China	MG-2100	RIKEN	(2-4)	
	Kun	Kunming, China	MG-0529	RIKEN	(2-4,6-8)	
	Lzh	Lanzhou, China	MG-0507	RIKEN	(2-4,6-8)	
	Moscow	Moscow region, Chevnogolvka, Russia	MG-3056	RIKEN	(2-4)	*env*
	Novii	Novii settlement, USSR, Russia	MG-3012	RIKEN	(2-4)	
	Qiq	Qiqihare, China	MG-0992	RIKEN	(2-4,6-8)	
	Tac	Tacheng, China	MG-0611	RIKEN	(2-4,6-8)	
	Wuh	Wuhan, China	MG-0908	RIKEN	(2-4)	

[1]Samples sorted according to genetic relatedness. *spp.*, subspecies undetermined.

Table S7. Primers used for PCR to amplify segments of mA3 and MMTV related sequences.

Gene	Amplicon	Forward Primer	Reverse Primer	Size (bp)
mA3	Exon2	CCTTGTTCTGCTTGGGGTCACTCC	GGATTCAAGGTATGAGCCACCATGC	333, 868[a]
	Exons 3-4	GCTTCAACAGGGCTCAGAGTGC	GGTTTGGGAGGAGGGAGAAC	770
	Exons 2-4	CCTTGTTCTGCTTGGGGTCACTCC	GGTTTGGGAGGAGGGAGAAC	2439
	Exons 6-8	CCAGAGTTGGGTTAAGAGCGG	GAGGAAGATGGGCAACAGG	1842
	Near full length	CCTGATAGAATTCCTCTTTCTCCTGG	GGCTCGAGCTCTCAAGCGTAATCTGG AACATCTATGGGTAAGACATCGGGGGTCC	1188
Mtv env		GACCCTAGACCCCATCAAAG	GAGGAAGTTGGCTGTGGTC	1036, 2197[b]
		CCTGGAAAGGACCCGATGTC	GACACTCTCGGGAGTTCAACC	959, 2117[b]
Mtv sag, env		GACCCTAGACCCCATCAAAG	GACCCTCTGGAAAGTGAAGG	2002, 3161[b]
		CCAGAATGTCCACTAGGTGTC	GACCCTCTGGAAAGTGAAGG	1692
Mtv pol		GGAAGAACGAGGCTCAGAAGG	CCAGTGTGTCCTCTGATATGACC	2057
		GGAAGAACGAGGCTCAGAAGG	GATAAGACACCACATCACCCTG	1111

[a]Contains MLV LTR.

[b]Some *env* genes have a 1161 bp deletion.

Figure S1. Comparative analysis of the 5’ and 3’ LTRs of *Mpmv5*, *Pmv23*, *Mpmv7* and *Mpmv4C* for shared or 3’LTR-specific G>A mismatches.

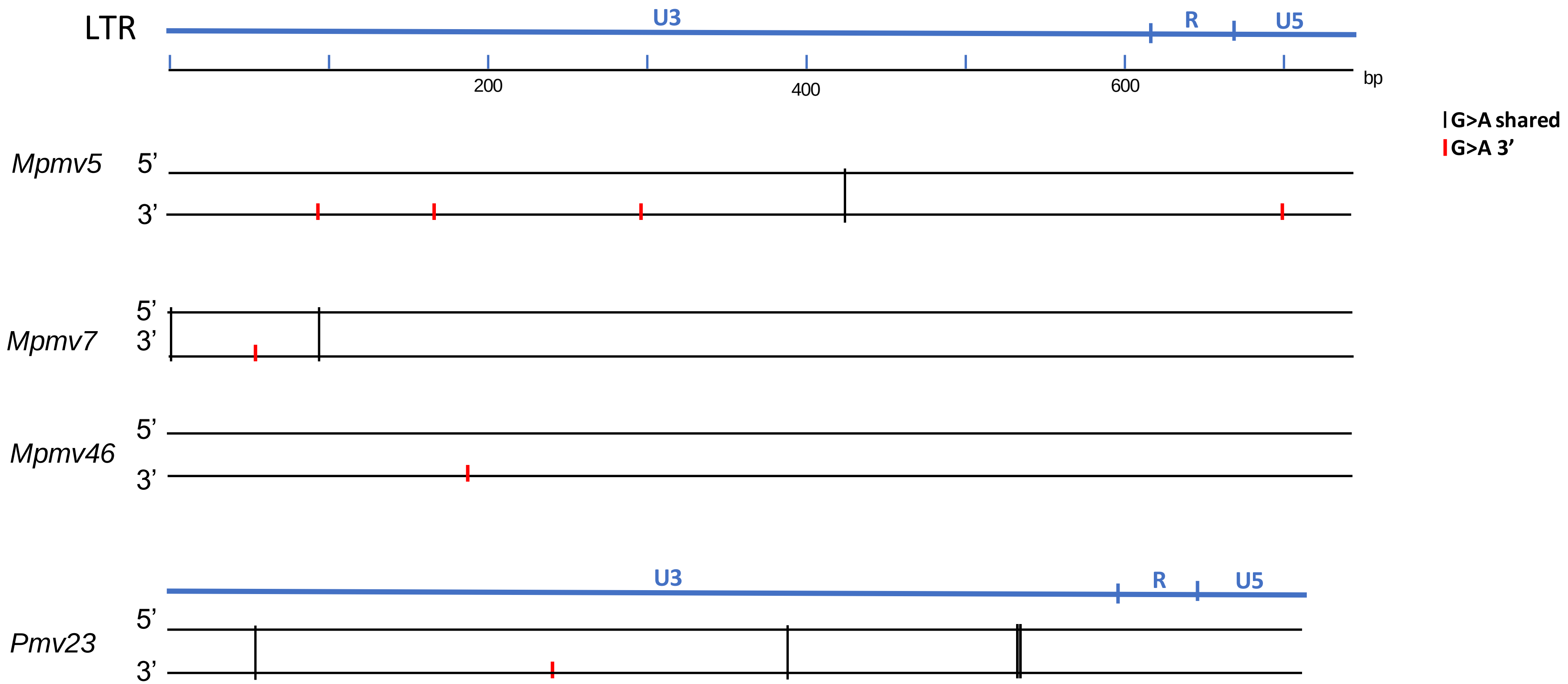

Figure S2. Sequence contexts of all mA3 hypermutations at the -1 and -2 nucleotide positions relative to minus strand targeted cytosines in individual *Mtvs*: **A)** *Mtvs* with moderate levels of hypermutation showing mA3^{B6}-like target preferences; **B)** heavily mutated *Mtv21* and *-61* which show mA3^{BALB}-like target preferences; **C)** 5' and 3' halves of *Mtv61*; **D)** shared G>A mutations in *Mtv* sets with different sequence contexts; **E)** the subset of *Mtv21* mutations with no other mutations within 3 nucleotide positions. Red bolding marks the most frequently used sequence context.

A ***Mtv17***

XXC \ XXC	A	C	G	T	
A	0	1	2	2	5 (6%)
C	1	1	0	6	8 (9%)
G	2	4	1	2	9 (10%)
T	1	24	4	36	**56 (75%)**
	4 (5%)	30 (34%)	7 (8%)	46 (53%)	87

Mtv57

XXC \ XXC	A	C	G	T	
A	4	3	3	10	20 (12%)
C	1	10	1	12	24 (14%)
G	3	2	1	2	8 (5%)
T	2	48	6	64	**120 (70%)**
	10 (6%)	63 (37%	11 (6%)	88 (51%)	172

Mtv62

XXC \ XXC	A	C	G	T	
A	0	1	2	5	8 (9%)
C	1	1	0	1	3 (3%)
G	1	3	0	4	8 (9%)
T	0	20	6	43	**69 (78%)**
	2 (2%)	25 (28%)	8 (9%)	53 (60%)	88

B ***Mtv21***

XXC \ XXC	A	C	G	T	
A	24	44	12	133	213 (23%)
C	24	77	5	108	216 (23%)
G	15	29	6	92	136 (14%)
T	29	139	38	174	380 (40%)
	92 (10%)	289 (31%)	61 (6%)	**501 (53%)**	943

Mtv61

XXC \ XXC	A	C	G	T	
A	15	12	10	48	85 (22%)
C	9	23	2	44	78 (20%)
G	5	12	8	44	69 (18%)
T	89	55	14	74	151 (39%)
	37 (10%)	102 (27%)	34 (9%)	**210 (55%)**	383

C ***Mtv61 (1-4473bp)***

XXC \ XXC	A	C	G	T	
A	10	7	4	5	26 (24%)
C	3	7	0	7	17 (13%)
G	2	6	6	8	22 (21%)
T	5	16	8	13	**42 (39%)**
	20 (19%)	36 (34%)	18 (17%)	33 (31%)	107

Mtv61 (4474-7923bp)

XXC \ XXC	A	C	G	T	
A	5	5	6	43	59 (21%)
C	3	16	2	37	61 (22%)
G	6	6	2	36	47 (17%)
T	5	39	6	61	109 (39%)
	16 (6%)	66 (24%)	16 (6%)	**177 (64%)**	276

D ***Mtv17,Mtv57,Mtv62***

XXC \ XXC	A	C	G	T	
A	0	0	1	0	1 (2%)
C	0	1	0	2	3 (5%)
G	2	2	0	1	5 (9%)
T	0	17	3	26	**46 (84%)**
	2 (4%)	20 (36%)	4 (7%)	29 (53%)	55

Mtv21,Mtv61

XXC \ XXC	A	C	G	T	
A	4	7	3	47	61 (20%)
C	4	22	0	43	69 (22%)
G	2	6	1	40	49 (16%)
T	5	49	6	73	133 (43%)
	15 (5%)	84 (27%)	10 (3%)	**203 (65%)**	312

E ***Mtv21*, standalone**

XXC \ XXC	A	C	G	T	
A	24	27	12	131	194 (32%)
C	10	7	0	31	48 (8%)
G	16	11	5	85	117 (19%)
T	28	4	38	176	246 (41%)
	78 (13 %)	49 (8%)	55 (9%)	**423 (70%)**	605

Figure S3. Sequence variants of mA3 exons 2-4 in wild mice. At the top is a diagram of the first four mA3 exons showing the locations of sites that distinguish mA3^{BALB} and mA3^{B6}. The chart shows amino acid residues at these and two additional sites for 19 wild mice.

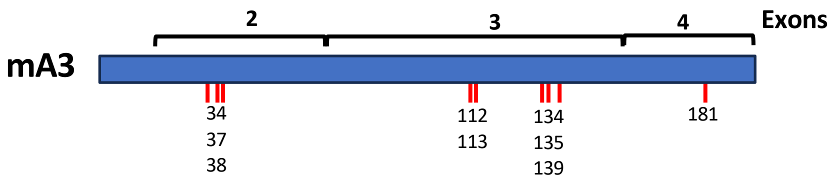

	Variable Sites											
Type	33	34	37	38	112	113	134	135	136	139	181	184	Mice
mA3^{B6}	L	G	K	G	I	V	V	Q	D	T	R	T	*M.m.domesticus*-Lewes
8	.	R	.	.	.	.	.	R	V	.	.	.	*M.m.musculus*-BLG2,MYL; *M.musculus*-Grozny
15	.	R	.	.	V	L	I	R	.	N	K	.	*M.musculus*-Jin,Wuh,Novii,Gui
mA3^{BALB}	.	R	I	D	V	L	I	R	.	N	K	.	*M.m.domesticus*-JJD,ABUR,SK,PERC; *M.musculus*-Moscow,Dal; *M.m.musculus*-Vej,Skive,Krk1,Donetsk,Akt

Datafile S1. Alignment of *Mtv21* with *Mtv1* showing mismatched nucleotides, with G>A mismatches in red.

```
Mtv1    CTTGCGGTTCCCAAGGTTTAAGTAAGTTCATGGTTACAAACTGTTCTTAAAACAAGGATGTGAGACAAGTGGTTTCCTGACTTGGTTTGGTATCAAATGT
Mtv21   ............T.AA.....AC..A.....A............. .........AA....A.A....A.........A ......... AA ...... A.

Mtv1    TTTGATCTAAGCTCTGAGTGTTCTATTCTCCTATGTTCTTTTGGAACTTATCCAAGTCTTATGTAAATGCTTATGTAAACCATGATATAAAAGAGTGCTG
Mtv21   ...A...........A.........AC...............AA..TC......T............................A.......... A .... A

Mtv1    ATTTTTTGAGTAAACTTGCAACAGTCCTAATATTCACGTCTCGTGTGTTTGTGTCTGTTCGCCATCCCGTCTCCGCTCGTCACTTATCCTTCACTTTCCA
Mtv21   .......A.A....G..A..........C..C...C..............A............................................C....

Mtv1    GAGGGTCCCCCCGCAGACCCCGGTGACCCTCAGGTCGGCCGACTGCGGCAGCTGGCGCCCGAACAGGGACCCTCGGATAAGTGACCCTTGTCTCTATTTC
Mtv21   A..........A..A.......C.......................AA...........................A.A.....................

Mtv1    TACTATTTGGTGTTTGTCTTGTATTGTCTCTTTCTTGTCTGGCTATCATCACAAGAGCGGAACGGACTCACCATAGGGAGCTGCAGTCCCGCCTACGGAG
Mtv21   ..............C.......T........A.............................A.........C.........................

Mtv1    AAGAGGTAGGTTACGGTGAGCCATTGGAAATGGGGGTCTCGGGCTCAAAAGGGCAGAAACTCTTTGTTTCTGTTTTACAAAGGCTCCTCTCAGAGAGGGG
Mtv21   G...................................................A........C..................T..............

Mtv1    TCTTCATGTGAAAGAGAGTAGTGCAATAGAATTTTATCAGTTTCTAATAAAGGTTTCTCCTTGGTTTCCCGAAGAAGGAGGATTAAATTTACAAGATTGG
Mtv21   .........A...A...A....A.....A.G...................A.....C....A...C..T.....G.A...........G...A.....

Mtv1    AAAAGGGTAGGAAGAGAAATGAAGAGGTACGCAGCGGAACATGGGACAGATAGCATACCAAAACAGGCTTACCCCATTTGGCTTCAGTTGAGAGAGATAC
Mtv21   .............A.A....A..A.A...........G...AAA...A...A........GG..A.........................A .... A .. T

Mtv1    TGACAGAGCAATCAGACTTGGTTTTGCTATCCGCAGAAGCCAAATCTGTTACCGAAGAAGAATTAGAGGAAGGTTTAACCGGACTACTATCGACAAGTTC
Mtv21   .A...A.A.............C..A.................G.....G...A..A..A.....A.AA................G..G..A....A...

Mtv1    ACAAGAAAAAACTTATGGGACCAGGGGAACAGCATATGCAGAAATAGATACAGAGGTAGATAAGCTGTCTGAACATATTTATGATGAACCATATGAAGAA
Mtv21    .......................AAAA....A........A.....A.....A.A.C.A.C...T....CA...........A..A........-..A..

Mtv1    AAGGAGAAGGCAGATAAAAATGAGGAAAAGGACCATGTTAGAAAAGTAAAGAAGATAGTACAAAGAAAAGAAATTAGTGAGGGTAAGAGAAAAGAGAAGG
Mtv21   ..AA.A......A........A.AA...AA.........A....A....A..AG.........A....A...A....A.......A.A....A.A..AA

Mtv1    ATCAAAAGGCCTTTTTAGCCACAGATTGGAACGATGATGACCTGTCCCCTGAGGATTGGGATGATTTAGAGGAACAAGCGGCACATTATCATGATGATGA
Mtv21   ......................A...AA...A..A..A....A......A.AA..............A.AA......AA...........A..A..A.

Mtv1    TGAGCTAATCCTTCCAGTAAAAAGGAAGGTGGTTAAGAAAAAACCTCAGGCACTCAGAAGAAAACCCCTGCCTCCGGTGGGTTTTGCAGGAGCGATGGCA
Mtv21   .A..............A....................A...........AA....T.....G. ................... C. ............... G

Mtv1    GAGGCCAGGGAAAAGGGAGATTTGACTTTTACGTTTCCTGTAGTTTTTATGGGAGAAAGTGATGATGATGATACGCCTGTTTGGGAACCGCTGCCATTAA
Mtv21   .....................C............................AAA.A.C.........A..A.............AAA...............

Mtv1    AAACCTTAAAGGAATTGCAATTGGCGGTTAAGACCATGGGACCATCTGCTCCATACACCTTGCAGGTGGTAGACATGGTAGCTAGTCAATGGCTTACCCC
Mtv21   .G................A.....A.........................G...................A....AA.........G.......T..

Mtv1    GAGTGATTGGCACCAAACAGCCAGAGCTACCTTATCCCCTGGGGATTATGTTTTATGGAGGACTGAATATGAAGAGAAAAGTAAAGAAACAGTACAAAAA
Mtv21    A.....................A................AAAA............AA.AA...A.....-..A.A.........A..............

Mtv1    GCCGCAGGCAAGCGAAAGGGCAAGGTCTCCCTTGACATGTTACTGGGGACTGGTCAGTTTCTGTCCCCTTCCTCTCAGATCAAATTATCTAAGGATGTCT
Mtv21   ......AA.....A.........A...........................C..A..C...........T...................AA......

Mtv1    TAAAAGATGTCACCACAAATGCTGTGTTAGCATGGAGAGCAATTCCGCCTCCTGGAGTCAAGAAGACTGTATTAGCAGGGTTAAAACAGGGAAATGAAGA
Mtv21   ..........T.....C............A.......G.....................T..A.............A..AAA.......AAA....A..A.

Mtv1    ATCTTATGAAACCTTCATTTCAAGACTTGAGGAAGCTGTTTATAGAATGATGCCAAGAGGAGAGGGATCAGATATATTGATCAAACAACTGGCGTGGGAA
Mtv21   .......A....T...........G..CA.AA..........C.A...................A..G...A.......A.........T.....AAA..

Mtv1    AATGCAAATTCATTGTGTCAGGATCTTATCCGTCCAATACGCAAAACAGGAACTATACAGGATTATATTCGTGCTTGCCTTGACGCTTCTCCCGCAGTTG
Mtv21   ...................AA....C......................AA.........AA....C..........A...AA.T.........A......

Mtv1    TTCAGGGTATGGCATATGCAGCAGCCATGAGAGGGCAAAAGTATTCTACTTTTGTAAAGCAAACATATGGTGGGGGAAAAGGGGGTCAAGGATCAGAAGG
Mtv21   .................A..A.......A.A.AAA........................A..G.........AAAAA....AAA......AA....A....

Mtv1    GCCAGTTTGTTTCTCCTGTGGCAAGACAGGACACATCAAAAAAGACTGTAAGGAGGAGAAGGGCTCTAAAAGGGCTCCTTCTGGGCTTTGTCCCAGATGT
Mtv21   ...G........T....A...T...T..AA.............A...A...AA..A.A..A...A.C.....A..C...C..A..... G. ......... A

Mtv1    AAGAAAGGCTACCACTGGAAGAGTGAGTGTAAATCTAAATTTGACAAAGATGGGAATCCACTTCCTCCTTTAGAAACTAATACTGAAAATTCAAAAAACT
Mtv21   ..A..G..........AA..A...A.A...............A.....A..AAA........C.....C...A...A....G..A..............

Mtv1    TGTAAAGGGGCAGTCCCCTAGCCCCACTCAAAAGGGGGATGGAGTTAAGGGCTCAGGATTAAATCCTGAAGCCCCACCTTTCACAATACATGATTTGCCT
Mtv21   ........................................AA.A....AAA..........................................A........

Mtv1    CGAGGCACCCCTGGAAGTGCAGGTTTAGACCTGTCATCACAGAAGGATTTGATCCTCTCTCTAGAAGATGGAGTATCATTGGTACCCACCTTAGTGAAAG
Mtv21   .A..C.......AA.............A.............A..A.....A............A..A..AA.A.......A.............A....

Mtv1    GTACCCTCCCTGAAGGGACTACTGGATTAATAATAGGTAGAAGTTCCAATTATAAAAAGGGACTCGAGGTTTTACCAGGAGTCATTGACTCCGATTTCCA
Mtv21   ..........A..AAA......AA..........A...A......T...........AAA....A.A...........G......A.....A.......
```

```
Mtv1   AGGAGAAATCAAGGTTATGGTTAAGGCCGCAAAAAATGCGGTCATCATTCACAAAGGAGAAAGAATAGCACAACTTCTGTTGCTGCCGTATTTAAAATTG
Mtv21  .AA.A.............AA.A........T..............T.........AA.A...A............G............ A .......... A

Mtv1   CCCAATCCTATAATCAAGGAAGAACGAGGCTCAGAAGGCTTCGGATCAACAAGTCATGTACATTGGGTGCAGGAAATAAGTGATTCCAGACCCATGCTTC
Mtv21  .................AA..A...........A........AA...............G.....A..A..AA........A..................

Mtv1   ATATTTCCTTGAATGGAAGAAGATTCCTCGGTCTCTTGGATACCGGGGCAGATAAAACTTGTATAGCAGGCAGAGACTGGCCAGCTAATTGGCCTATTCA
Mtv21  ......A...A...AA..A..A.......A........A.......AA..A.........A.......AA..A.A...................C.....

Mtv1   CCAAACTGAAAGTTCTCTTCAAGGTTTAGGCATGGCCTGTGGGGTGGCGCGTAGTAGTCAGCCACTCCGCTGGCAACATGAGGATAAATCAGGGATTATA
Mtv21  .......A...A..........A.....AA...A......AAAA.A.........................AA......A.AA........AAA......

Mtv1   CATCCCTTTGTGATCCCTACACTGCCCTTTACCTTGTGGGGAAGAGACATTATGAAAGAGATAAAGGTCAGGTTAATGACTGACTCACCAGATGATTCAC
Mtv21  .....T.....A..............T..C.....A.AAAA..A.A.......A...A.A..........AA.....A..CA........A..A.C..T.

Mtv1   AGGATTTATGATAGGGGCCATTGAGAGCAATCTCTTTGCAGACCAAATATCTTGGAAATCAGACCAGCCTGTATGGCTTAATCAATGGCCCCTTAAACAA
Mtv21  .AA......A............A.A...............A............AA......A........A.............................

Mtv1   GAAAAGTTACAGGCTTTACAACAGTTAGTGACAGAACAATTACAACTGGGCCACTTAGAAGAGAGCAATAGCCCTTGGAATACGCCTGTTTTTGTCATTA
Mtv21  A...................G..A.....A...A....................C..A..A.A.............AA.....A................

Mtv1   AAAAGAAGTCAGGAAAATGGAGGCTGTTACAAGACCTACGTGCAGTTAATGCCACAATGCACGATATGGGAGCATTACAACCAGGCTTGCCGTCCCCTGT
Mtv21  ....A..A...AA.....AA............A........A..........T.........A....AAA.A......................T.....

Mtv1   AGCAGTCCCTAAAGGATGGGAAATAATCATAATAGATCTACAAGATTGCTTCTTTAATATAAAACTGCATCCTGAAGATTGTAAAAGATTTGCTTTTAGT
Mtv21  .............AA..AAA..............A........A....T.................A......A..A.........A.............

Mtv1   GTGCCCTCCCCTAATTTTAAGAGACCCTATCAAAGATTCCAGTGGAAAGTTTTGCCCCAGGGTATGAAAAATAGCCCTACTTTATGTCAAAAATTTGTAG
Mtv21  .....T..T........C..A..G..T.......A......A.AA....C...............A..............................A..A

Mtv1   ATAAAGCTATATTGACTGTAAGGGATAAATATCAAGACTCATATATTGTGCATTACATGGATGACATTCTTTTGGCACACCCATCAAGATCCATTGTTGA
Mtv21  .C.........C.A.......AAA...........A......................AA..A.............T.......... A.G. ..... CA.

Mtv1   TGAAATACTTACTTCCATGATACAGGCCCTTAACAAACATGGCCTTGTAGTATCCACAGAGAAGATTCAAAAATATGATAATCTCAAATATTTGGGAACT
Mtv21  .A................A...........................A...........A.A..A............A................AAA....

Mtv1   CATATACAGGGTGATGTGGTGTCTTATCAAAAATTACAGATTAGGACAGATAAATTAAGAACCTTAAATGATTTCCAAAAGCTGCTAGGAAATATTAATT
Mtv21  T........A..A..A......................A....AA...A.........A..........A.................AA...........

Mtv1   GGATACGTCCTTTCTTAAAATTAACTACGGGAGAGTTAAAACCTCTCTTTGAAATCCTTAACGGAGACTCTAATCCTATCTCAATAAGAAAACTTACTCC
Mtv21  AA....A.......C..........C..AAA.A.................A..........TAA.A.............T.......A............

Mtv1   TGAGGCATGCAAAGCTCTTCAATTGGTAAATGAAAGACTATCTATCGCTCGGGTAAAGAGGCTAGATTTATCACGGCCTTGGTCTCTATGTATATTAAAG
Mtv21  .A...........A..........AA.....A...A........C.....AAA....A.AA...A..C......A............. C.A ........ A

Mtv1   ACTGAATATACCCCCACAGCATGCCTCTGGCAAAATGGAGTCCTAGAATGGATACATTTGCCTCATATTTCACCAAAAGTAATTACTCCTTATGATATCT
Mtv21  ...A.......T.......................CAA.A..A..A...AA...........................A..............A...C..

Mtv1   TTTGTACACAACTTATTATTAAGGGCCGACACCGCTCTAAGGAATTATTTAGTAAAGACCCTGATTATATTGTTGTGCCCTATACCAAAGTTCAATTCGA
Mtv21  ...........................A............AA.........A....A.....A.....G..A.C......................... TA.

Mtv1   TCTCCTATTACAAGAAAAGGAAGATTGGCCTATTTCTTTATTAGGGTTCCTGGGAGAGGTTCATTTCCATCTTCCAAAAGACCCTTTGCTTACATTTACC
Mtv21  .............A....AA..A..........C..C..............AAA.A.......G...............A.....C..............

Mtv1   CTACAAACTGCCATTATTTTTCCTCACATGACCTCTACCACACCACTAGAGAAAGGAATTGTGATTTTTACGGACGGGTCAGCAAATGGCCGTTCGGTAA
Mtv21  .............................A..................A.A...AA......A........AA....T...A.............A....

Mtv1   CATATATACAAGGAAGGGAGCCTATAATTAAAGAAAATACACAAAACACAGCCCAACAGGCTGAAATTGTGGCAGTCATTACAGCCTTTGAGGAAGTGAG
Mtv21  ................................A....C..............A.........A..........................A.AA..A.A.A

Mtv1   TCAATCCTTTAATTTGTATACTGATTCTAAATATGTGACTGGGTTGTTTCCCGAAATCGAAACTGCAACTTTGTCACCCAGAACAAAAATTTACACAGAA
Mtv21  ....C.................A.............A...A...........A.....A.....A....C..........A................A..

Mtv1   CTGAGACATTTACAAAGGTTAATCCACAAGAGACAAGAAAAATTTTACATTGGTCATATCAGAGGACACACTGGACTTCCCGGTCCTTTGGCACAGGGAA
Mtv21  ..A.A...........A............A.A....A...............A........A.AA.......AA...............AA.........

Mtv1   ATGCCTATGCGGATTCCTTAACAAGAATTCTGACCGCTTTAGAGTCAGCTCAAGAAAGCCACGCACTACATCATCAAAATGCCGCGGCGCTTAGGTTTCA
Mtv21  ..........A.....T....................C...A...........A................C.............................

Mtv1   GTTTCACATCACTCGTGAACAAGCACGAGAAATAGTAAAACTATGTCCAAATTGCCCCGACTGGGGACATGCGCCACAACTAGGAGTAAACCCCAGGGGC
Mtv21  ................A.....A...A.A....TA.......................A...AAAA................AA.A..............

Mtv1   CTTAAGCCCCGAGTTCTATGGCAAATGGATGTTACTCATGTCTCAGAATTTGGAAAATTAAAGTATGTACATGTGACAGTAGATACCTATTCTCATTTTA
Mtv21  ..........A.A......AA.....AA.......C.........A.....AA...................A.A......A..................

Mtv1   CTTTCGCTACCGCCCGAACGGGCGAAGCAACCAAAGATGTGTTACAACACTTGGCTCAAAGCTTTGCATACATGGGCATTCCTCAAAAAATAAAAACAGA
Mtv21  ...............A.......A...........A..A.............AA .................. A ...................... A.
```

```
Mtv1   TAATGCCCCTGCATATGTGTCCCGTTCAATACAGGAATTTCTGGCCAGATGGAAAATATCTCACGTCACGGGGATCCCTTACAATCCCCAAGGACAGGCC
Mtv21  ..................................AA............A..AA................AAAA..................AA.......

Mtv1   ATTGTTGAACGAACGCACCAAAATATAAAGGCACAGCTTAATAAACTTCAAAAGGCTGGAAAATACTATACACCCCACCATCTATTGGCACATGCTCTTT
Mtv21  ......A...A........................................AA.................T........A............

Mtv1   TTGTGCTGAATCATGTAAATATGGACAATCAAGGCCATACGGCGGCCGAAAGACATTGGGGTCCAATTTCAGCCGATCCAAAACCTATGGTTATGTGGAA
Mtv21  .......A......A.......AA...............A......A...A..................G...A.............AA ....... AA..

Mtv1   AGATCTTCTCACAGGGTCCTGGAAAGGACCCGATGTCCTAATAACAGCCGGACGAGGCTATGCTTGTGTTTTTCCACAGGATGCCGAATCACCAATTTGG
Mtv21  .A.................AA.........................AA..A...........A............AA.....A.............

Mtv1   GTCCCTGACCGATTCATCCGACCTTTCACTGAGCGGAAAGGATCGACGCCCACGCCTAGCACTGCGGAGAAAACGCCGCCGCGAGATGAGAAAGATCACC
Mtv21  .....C.....G.......A......T.......AA...AA...A........A...G..G..A.AA.A.............A.A..A.A .... A ... A.

Mtv1   AAGAAAGTCCGGAGAATGACCCTAGACCCCATCAAGGAGAAGACGGCTTGGCAACAGCTGCAGGCGTTGATCTCCGAAGCGGAGGAGGTTCTTAAAACCT
Mtv21  ..A.......AA.A...A.AT...A..........AA.A..A.......AA.....A...........A......A....AA.AA.AA...........

Mtv1   CACAAACTCCCCAAACCTCTTTGACCTTATTTCTTGCTTTGTTGTCTGTCCTCGGCCCCCTGCCTGTGACCGGGGAAAGTTATTGGGCTTACCTACCTAA
Mtv21  ......................A......................T.A...........T.......A...AAAA...A.........C...........

Mtv1   ACCACCTATTCTCCATCCCGTGGGATGGGGAAGTACAGACCCCATTAGAGTTCTGACAAATCAAACCATGTATTTGGGTGGTTCGCCTGACTTTCACGGG
Mtv21  ......A............A.AAA..AAAA.......A.........A.....A.......................G. ......... A ...... TAA.

Mtv1   TTTAGAAACATGTCTGGTAATGTACATTTTGAGGGGAAGTCTGATACGCTCCCCATTTGCTTTTCCTTCTCCTTTTCTACCCCCACGGGCTGCTTTCAAG
Mtv21  ....A................A........A.AAAA......A....A............C.....................T. ....... A ...... A

Mtv1   TAGATAAGCAGGTATTTCTTTCTGATACACCCACGGTTGATAATAATAAACCTGGGGGAAAGGGTGATAAAAGGCGTATGTGGGAACTTTGGTTGACTAC
Mtv21  ..A....A..A............A..............A..............AAAAA...AA..A.........A.....AAA......AA..A.....

Mtv1   TTTGGGGAACTCGGGGGCCAATACAAAACTGGTCCCTATAAAAAAGAAGTTGCCCCCCAAATATCCTCACTGCCAGATCGCCTTTAAGAAGGACGCCTTC
Mtv21  C..AAAA.....A.................A..............A..A..........................A...........A..AA........

Mtv1   TGGGAGGGAGACGAGTCTGCTCCTCCACGGTGGTTGCCTTGCGCCTTCCCTGACCAGGGGGTGAGTTTTTCTCCAAAAGGGGCCCTTGGGTTACTTTGGG
Mtv21  .AAA.AAA.A..A.A.................A...................A..........A.A ...................... AAAC. .... AAA

Mtv1   ATTTCTCCCTTCCCTCGCCTAGTATATAGATCAGTCAGAACAAATTAAAAGCAAAAAGGATCTACTTGGAAATTATACTCCCCCTGTCAATAAAGAGGTTCA
Mtv21  ........................A.G..A....A...A.T..........A......AA.....T..AA................A........A.A.....

Mtv1   TCGATGGTATGAAGCAGGATGGGTAGAACCTACTTGGTTCTGGGAAAATTCTCCTAAGGATCCCAATGATAGAGATTTTACTGCTCTAGTTCCCCATACA
Mtv21  ..A..A....A..A..AA..AAA..A.............T.AAA.............AA....A...A...A.A.........................

Mtv1   GAATTGTTTCGCTTAGTTGCAGCCTCAAGACATCTTATTCTCAAAAGGCCAGGATTTCAAGAACATGAGATGATTCCTACATCTGCCTGTGTTACTTACC
Mtv21  A...........................A.................A....AA.......A.....A.A..A........................... T.

Mtv1   CTTATGCCATATTATTAGGATTACCTCAGCTAATAGATATAGAGAAGAGAGGATCTACTTTTCATATTTCCTGTTCTTCTTGTAGATTGACTAATTGTTT
Mtv21  .................AA................A.....A.A..A.A.AA........................C....A..A...A..........

Mtv1   AGACTCTTCTGCCTACGACTATGCAGCGATCATAGTCAAGAGGCCGCCATATGTGCTGCTACCTGTAGATATTGGTGATGAACCATGGTTTGATGATTCT
Mtv21  .A.T............A.....A....A......A....A............A...........A..A.....AA.A..A......A....A..A.....

Mtv1   GCCATTCAAACTTTTAGGTATGCCACAGATTTAATTCGAGCTAAGCGATTTGTCGCGGCCATTATTCTGGGCATATCTGCTTTGATTGCTATTATTACTT
Mtv21  ..........C....A......T...A.........A........A.........A............AA............A...........C....

Mtv1   CCTTTGCTGTAGCTACTACTGCCTTAGTTAAGGAGATGCAAACTGCTACGTTTGTTAATAATCTTCATAGGAATGTTACATTAGCCTTATCTGAACAAAG
Mtv21  ..................T...T........AA.A...........C......A.......C.......AA...A................... A ..... A

Mtv1   AATAATAGATTTAAAATTGGAAGCTAGACTTAATGCTTTAGAAGAAGTAGTTTTAGAGTTGGGTCAAGATGTGGCAAATTTAAAGACCAGAATGTCCACT
Mtv21  .......A..........AA......A.............A..A..A..A.....A....AAA....A..A.A.....C.....A....A..........

Mtv1   AAGTGTCATGCAAATTATGATTTTATCTGCGTTACACCACTACCATATAATGCTTCTGAGAGCTGGGAAAGGACCAGAGCTCATTTACTGGGCATTTGGA
Mtv21  ..A................A.........A.A........T.................A.A....AAA...AA....A........... T.AA ..... AA.

Mtv1   ATGACAATGAGATTTCATATAACATACAAGAATTAACCAACCTAATTAGTGATATGAGCAAACAACATATTGACGCAGTGGACCTTAGTGGCTTGGCTCA
Mtv21  ..A.....A.A........C.....T...A................C.A.A....A.A.............A..A....AA....C.............

Mtv1   GTCTTTTGCCAACGGAGTGAAGGCTTTAAATCCATTAGATTGGACACAATATTTCATTTTTATAGGAGCTGGAGCCCTGCTTTTAGTCATTGTGCTTATG
Mtv21  ...C........TAA.A.A..................A...AA.....................AA.A..AA................... AA.A .... A

Mtv1   ATTTTCCCCATTGTTTTCCAGTGCTTTGCGAAGAGCCTTAACCAAGTGCAGTCAGATCTTAACGTGCTTCTTTTAAAAAAGAAAAAAGGGGGAAATGCCG
Mtv21  ...........................A.A..A..............A..A...A.............................................

Mtv1   CGCCTGCAGCAGAAATGGTTGAACTCCCGAGAGTGTCCTACACTTAGGAGAGAAGCAGCCAAGGGGTTGTTTCCCACCAAGGACGACCCGTCTGCGTGCA
Mtv21  .....A.....A........A.......A.A...............AA.A.A...........................AA..............CA..

Mtv1   CGCGGATGAGCCCATCAGACAAAGACATACTCATTCTCTGCTGCAAACTTGGCATAGCTCTGCTTTGCCTGGGGCTATTGGGGGAAGTTGCGGTTCGTGC
Mtv21  .A.AA..A.........A.....A.......................................................AAAAA................
```

```
Mtv1   TCGCAGGGCTCTCACCCTTGACTCTTTTAATAACTCTTCTGTGCAAGATTACAATCTAAACGATTCGGAGAACTCGACCTTCCTCCTGGGGCAAGGACCA
Mtv21  ...................A............G.............A..............A....AA.A.....A............AA....AA....

Mtv1   CAGCCAACTTCCTCTTACAAGCCACACCGACTTTGTCCTTCAGAAATAGAAATAAGAATGCTTGCTAAAAACTATATTTTTACCAATGAGACCAATCCAA
Mtv21  .G........................T.A.T...........A.....A......A...............T...............A.A..........

Mtv1   TAGGTCGATTATTAATCATGATGTTAAGAAATGAATCTTTGTCTTTTAGCACTATATTTACTCAAATTCAAAGGTTAGAAATGGGAATAGAAAATAGAAA
Mtv21  ..A...A...........C........A....A....GC.AC.......T.....T........T.......A....A....AAA....A......A...

Mtv1   GAGACGCTCAACCTCAGTTGAAGAACAGGTGCAAGGACTAAGGGCCTCAGGCCTAGAAGTAAAAAGGGGAAAGAGGAGTGCGCTTGTCAAAATAGGAGAC
Mtv21  A.A.............A.CA..A....A......AA....TTAA..A........A..A......AAAA...A.AA.A.ATAT..A.T. ......AA.A..

Mtv1   AGGTGGTGGCAACCAGGGACTTATAGGGGACCTTACATTTACAGACCAACAGACGCCCCGTTACCATATACAGGAAGATATGATTTAAATTTTGATAGGT
Mtv21  .AA.AA.AA......AAA.......AAAA.........C....A.......A.T..T..A............AA..A....A.C.........A...A..

Mtv1   GGGTCACAGTCAACGGCTATAAAGTGTTATACAGATCCCTCCCCTTTCGTGAAAGGCTCGCCAGAGCTAGACCTCCTTGGTGTGTGTTGTCTCAAGAAGA
Mtv21  .......C.......A.................A.A............A.A...AA.....T.A.....A........AA...A.A..AA.....A..A.

Mtv1   AAAAGACGACATGAAACAACAGGTACATGATTATATTTATCTAGGAACAGGAATGATACATT
Mtv21  ....A..A....A........A......A..............AA....AA...A.-.....
```

Datafile S2. Sequence mismatches in the genomic sequences flanking *Mtv21* in the NZO/HILtJ genome relative to the orthologous segment in the B6 reference genome.

```
NZO   TTTTAATATTTTGTATCTATTACCTTTCTCTCTCCATTTTCAAAATTTGACCAAACGTTTCTGATATTTGCAAAATTTACATTAGTTGTAATTAATATTGGAGAAAGATTCATGTGCTTT
B6    ........................................................................................................................

NZO   ACCAAGGAGCCTGCATTCTTTAATTATTAGTACAGTATTCTGTAGATCTCTTTATTTAACTTGCAAACATGGTGATGTTTTACACCGAGGTTCTTTATGTTTATATGAATGCCTGAACAC
B6    ........................................................................................................................

NZO   TGATGAAAGTATAGTATTGAAGTTACTAGCTATAAGTGTGTTAAGATTAATCTATAGGGAAACATCTAATATAATGCATTTAAATAGGTGGGTATACACATGGTTTTTGCATATGTATTT
B6    ........................................................................................................................

NZO   TCTCTTGGTGGATTATTTCATTTATGAGCGTGATATGATATTCTATATAACTTATGACTAGTTTATCATGAAAATAATTTTTGAGATATTAAAATAGCTATGCCTGATTGTTTTCTTGTT
B6    ........................................................................................................................

NZO   CTACTTTCACAAAGTAATTTTTATATTCTTTTTTAAGAAGAACTTTTTCATGTTGAGGTATGTCTCCTGAAGATATCAAAATTATGAATTCACCCCTGATCCAATGTTTCAGCCTGTGTC
B6    ........................................................................................................................

NZO   CTTTTATTTGAAAAATTAAAATCTTAATATTCAGAAGTATTATGAAAGATATGCCTAAATTTCTGGCATTATGTAGATTTAGTGACATTTTTTTCAGACCTTGTCTCTTTAACTAACTTG
B6    ........................................................................................................................

NZO   ACATTGTTTATTTAGCTCCTTGACTTCAAAGGTGTGCTTGGACTTCTTTTGAATCTAAACTCTATCCTCTCTAGGATGCTGACTTGGGGAAAATAAATCTTTTAGCATGTTTTTTTATTA
B6    ........................................................................................................................

NZO   TGGAAACATTTTATTTCTGTTCCAATTATATAGCTTTGCTGGGTATAATAGTATGAGCTGACATTTGTAATGTTTCCTGACTCAGAATACATCTTTTTCGGCACCAGCTGGCTTTAAATA
B6    ........................................A...............................................................................

NZO   TTTCCATGAAAGAATCATATATTATTCTATTGATCTATTTATGTGACTTGACATTTTTCTTTTATACCTTTCAATATATTTATTTACTCTAGGTTTTAAACGTGTTAACTGAAATATGTT
B6    ........................................................................................................................

NZO   ATGCGAGTTTCTGTTCTAGTCCTGTTGATTTGGTGTTCAGTAAGCCTTTTATTCAACCTGAATAGGTATCTTTTTCTCTGGGGTTTGTAGATTTTCTTGTATGATTTAATTGAAAATATT
B6    ...A....................................................................................................................

NZO   TTCTATGCCTTTATTGGAGAGTCCTCTCCTTCCCCTCTCCATAATTTGAAGACTTGATCTTTTCATGGTACCCCAAAGTTGTCCTATGTTCTATGTACACAAACATGTGCACGCACACAC
B6    ..................................................................................T.....................................

NZO   ACAAACACTATA --------------------TATATATATATATATATATATATATATATATACATACATATATAAAACCTCCTTCAGTCTAGACAGGCTGGTTCAGACCATCTGCCAC
B6    ............TATATATATATATACATATG......................C.................................................................

NZO   TGGGGCAGAGCCACCTGCTGTGCTGCTTCTGTGCTGCTTCTCATCCCTCTCCTTTGATATGATACTGTCTGCCACCTGCCTGAGGCCTAACCTGTTCTCCAAGGTTTCCTGAGTTTAAAG
B6    ........................................................................................................................

NZO   CAAACTGTGAAATTGGCGAATTTCTGCTAAAAGGCTGTTCTGATGACTTCATAAGAAACTTGGCGTGACTGGGGATGGGCTTCCCTCTTTATAAAGCGCAGATTATTATTAAAGATTTGA
B6    ........................................................................................................................

NZO   GCCTTGATCAGAATCCTGTCTTGGTTCCATCTCTCTCTCTTCCGCCTAGTTTCCTTTTCTTTCAGCTCCAAGATGCCTCCCAAGCTCGAACCCAGACATGAGTTATAATTATATATATAT
B6    ........................................................................................................................

NZO   ATATATATATATATATATATATATATATATATATATAT--AATTGATTAGTACTGAGAAATTCAACTGCTCTGCCCTATCTTCACATCAGATAATCTGATTTCCCCATGCTCTTACTCAT
B6    ......................................AA...................................................................C............

NZO   TGGCAAGCCTTTTTCTCTTAGCTTCTTTAATTGGCTCCTAGG   [ Mtv21 ]   CCTAGGTATTTCACTTTCATCATCATCTCACTTTGGCTTTTCCTTAGGAGTTTTTTTCTTTTTAA
B6    ....................................------                .................................................................

NZO   TGAAATATATATTCATACCTTGAAGTAGC-TTTTTTTATTCAAATCATTTGTTCTCATGAAGTACTATCATATCTTCAGTGACAGAGTGTTGTGTGAAGACAACATAATCATTATGTTAA
B6    .............................T..........................................................................................
```

```
NZO    ACAATTTCTCTGGAGTGTCTTCCAAGTTTTCCTCATTGGAAGCCATTACCATGGTATTAGTAATTTTGAAGGGAAATATTCTGACTTCTTTTTTTAAGTTTGTTTCTTTGTTGGGGACTT
B6     ........................................................................................................................

NZO    GTAAATCTAGATTGAGTCTTTCTTACTTTCTGTTAATGAAGTCACCCCCTCTCCCACTTGAGACTTTATAAAGTTCAGGTAATGAATAAGGCTTTATAGTTCTGAGGTGTCTTCCTCTGC
B6     ........................................................................................................................

NZO    AAGACTGGTATTCAAGATGACAAATAGTATTTTTAGTGTTTCTCCAAGAATCAGACACTATATGCCACTAAAATCACTAGCCAAATATCAGCAAAATCCTGCCTAGTACCTGCCTCCACT
B6     ........................................................................................................................

NZO    TTAATCATGTGTGGCTCCCACACGGGTCTCCTTCAGTGGATAGCTGGCCTTCCATAGCTCCATGCTTGCTGTGAGAATCTGAATGAATGCCTTATTGCCTACCATAGGTCATCTCTGGTC
B6     ........................................................................................................................

NZO    ACTTCCTGGCTCTGATGCTTTATGGAAAATTATAGAACTTCCTGTGCCTGGCCTCCTATCCTGGATCAGCTTGTTATGGCCTCCCTTTTCAATACATTACATACTTTTATACTTTAAAGA
B6     ........................................................................................................................

NZO    CTTTTACAAAGCCCTGATCATGTTGTTTAAATGAAGTACAAAATAAATTAGAAATATAATTATACGGTTTACGTAAGATATCAATTGTAATTGTTTTCAGAAAAGTCTACTATTTTAGAA
B6     ........................................................................................................................

NZO    TTAGTAGTTTTGTTGTTACTCTTACTTAAGCCTATCATATGATTTTAGGTTCTAGGTAAGTGAAAAATAAAATCCAGGCAGAATTTTAGCATAATATTTGCAAGTTAAACTGAATTCACT
B6     ........................................................................................................................

NZO    GGTTTCCTGACAGAAATTCAATTTTGAAATTCCTAGCAAAAATAAAGACATGTTTAAATGGAAAAAAAAAAACTTGGTATTTGTTTAATTTTCATTGAAGTTTCAAAGTATTTCTTTTGGC
B6     .............................................................-..........................................................

NZO    ATTGCTTTTAAGTATGATGGGTTCTATGCAAACTTTGAAATGCCTTTTTACTTCATATTTCAGAATGCGTGTGCCTATGCATTCATAATGGAGAGAGATGATAAAGGTTCTCAGTTGCAT
B6     ........................................................................................................................

NZO    TAAAACTAAACACAATATAGGCAGCTGCTGTGAGCTAGACTATTATAGTTTTATCCCGATATATAAAATGGAAAATGTTGAGGACATTAAAAACCTATTACCTAGTTCTGATTGCGTACC
B6     ........................................................................................................................

NZO    AAAGAGAGACTACTCTC ---- AAAAAAAAAAAAGTCCTGGAATACTGTGAAATATGATTCAGGCACAGCCATTCTCCTGTGCCTATTTCTTACTTCCTTTTAACAATATAGGATATGTG
B6     .................AAAAA..................................................................................................

NZO    AAAAATATCCGTGGATTTCTTTTACAAGCATTACTGTAGTA--TTTTTTTGTTGTTGTTTTTACTGATAGAGAATTTTAGCTATTAAATCTGTTATCAATGAAGAAGTTTTATTATCAGT
B6     .........................................TT.............................................................................

NZO    TGTCTCAGTCAGGGTTTGTATTCCTGCACAAAATATCAGGATCACGAAGCAAGTTGGGGAAGAAAGGTATTATTCCAGTTATACTTCCACATTGCTGTTCATCACCAAAGGAAGTCAGGA
B6     ....................................................................T......................................T............

NZO    TAGAAACTCACACAGGGCAGGGACTTGGAAGCAGGAGATGATTCAGA ----- TGGAGGGATGCTGCTTACTGGCTTGCTTCCCCTGGCTTGCTCAGCCTGCTTTCTTATAGAACCTAAGA
B6     ........................G............C.........GGCTG..............G..............................T.................C....

NZO    CTGCCAGTCCAACGATGGCACCACCCACAATTTGCTGGGCCCTCCCACCTTGATCACTAATTGAGAAAATGCCTTACAGCTGGATCTCATGGAGGCATTTCCTAAGGGAGGCACCTTTCT
B6     ..A.....................................................................................................................

NZO    CTGTGATAACTCCTGCTTGTTT
B6     ......................
```
